# A Multiomic Approach to Understand How *Pleurotus eryngii* Transforms Non-Woody Lignocellulosic Material

**DOI:** 10.3390/jof7060426

**Published:** 2021-05-28

**Authors:** Ander Peña, Rashid Babiker, Delphine Chaduli, Anna Lipzen, Mei Wang, Mansi Chovatia, Jorge Rencoret, Gisela Marques, María Isabel Sánchez-Ruiz, Teeratas Kijpornyongpan, Davinia Salvachúa, Susana Camarero, Vivian Ng, Ana Gutiérrez, Igor V. Grigoriev, Marie-Noëlle Rosso, Angel T. Martínez, Francisco J. Ruiz-Dueñas

**Affiliations:** 1 Centro de Investigaciones Biológicas Margarita Salas (CIB), Consejo Superior de Investigaciones Científicas (CSIC), 28040 Madrid, Spain; ander.pena@cib.csic.es (A.P.); rashid.babiker@cib.csic.es (R.B.); marisasr@cib.csic.es (M.I.S.-R.); susanacam@cib.csic.es (S.C.); atmartinez@cib.csic.es (A.T.M.); 2 Institut National de Recherche Pour L’agriculture, L’alimentation et L’environnement (INRAE), Aix Marseille Université, Biodiversité et Biotechnologie Fongiques, 13009 Marseille, France; delphine.chaduli@inrae.fr (D.C.); marie-noelle.rosso@inrae.fr (M.-N.R.); 3 Lawrence Berkeley National Laboratory, US Department of Energy (DOE) Joint Genome Institute (JGI), Berkeley, CA 94720, USA; alipzen@lbl.gov (A.L.); mwang@lbl.gov (M.W.); mrchovatia@lbl.gov (M.C.); vng@lbl.gov (V.N.); IVGrigoriev@lbl.gov (I.V.G.); 4 Instituto de Recursos Naturales y Agrobiología de Sevilla (IRNAS), Consejo Superior de Investigaciones Científicas (CSIC), 41012 Seville, Spain; jrencoret@irnase.csic.es (J.R.); gisela@irnase.csic.es (G.M.); anagu@irnase.csic.es (A.G.); 5 Renewable Resources and Enabling Sciences Center, National Renewable Energy Laboratory, Golden, CO 80401, USA; Teeratas.Kijpornyongpan@nrel.gov (T.K.); davinia.salvachua@nrel.gov (D.S.); 6 Department of Plant and Microbial Biology, University of California, Berkeley, CA 94720, USA

**Keywords:** *Pleurotus eryngii*, lignocellulose transformation, solid-state fermentation, wheat–straw, transcriptomics, proteomics, metabolomics, carbohydrate-active enzymes, oxidoreductases, lignin-modifying enzymes

## Abstract

*Pleurotus eryngii* is a grassland-inhabiting fungus of biotechnological interest due to its ability to colonize non-woody lignocellulosic material. Genomic, transcriptomic, exoproteomic, and metabolomic analyses were combined to explain the enzymatic aspects underlaying wheat–straw transformation. Up-regulated and constitutive glycoside–hydrolases, polysaccharide–lyases, and carbohydrate–esterases active on polysaccharides, laccases active on lignin, and a surprisingly high amount of constitutive/inducible aryl–alcohol oxidases (AAOs) constituted the suite of extracellular enzymes at early fungal growth. Higher enzyme diversity and abundance characterized the longer-term growth, with an array of oxidoreductases involved in depolymerization of both cellulose and lignin, which were often up-regulated since initial growth. These oxidative enzymes included lytic polysaccharide monooxygenases (LPMOs) acting on crystalline polysaccharides, cellobiose dehydrogenase involved in LPMO activation, and ligninolytic peroxidases (mainly manganese-oxidizing peroxidases), together with highly abundant H_2_O_2_-producing AAOs. Interestingly, some of the most relevant enzymes acting on polysaccharides were appended to a cellulose-binding module. This is potentially related to the non-woody habitat of *P. eryngii* (in contrast to the wood habitat of many basidiomycetes). Additionally, insights into the intracellular catabolism of aromatic compounds, which is a neglected area of study in lignin degradation by basidiomycetes, were also provided. The multiomic approach reveals that although non-woody decay does not result in dramatic modifications, as revealed by detailed 2D-NMR and other analyses, it implies activation of the complete set of hydrolytic and oxidative enzymes characterizing lignocellulose-decaying basidiomycetes.

## 1. Introduction

Lignocellulose is the most abundant component of Earth’s biomass, with over 180 billion tons produced annually [1]. Its abundance makes it a promising renewable feedstock alternative to non-renewable fossil resources. In this context, the lignocellulose biorefinery concept [2,3] is receiving considerable attention as a source of renewable chemicals, materials, and fuels for future sustainable development. The main challenges for developing this concept are related to the breakdown of the lignocellulose structure and to the optimized use of their main components, cellulose, hemicelluloses and lignin [4,5].

In nature, many basidiomycete fungi can grow on lignocellulosic materials. Their ability and efficiency to degrade lignocellulose largely depend on the repertoire of hydrolytic and oxidative enzymes able to act on the plant cell-wall polymers [6,7,8]. Among these organisms, white-rot basidiomycetes are the only ones capable of efficiently degrading lignin, the aromatic polymer that protects cell-wall polysaccharides from microbial and enzymatic attack. Due to this unique capability, white-rot fungi play a key role in carbon recycling in land ecosystems and provide valuable biotechnological tools for processing plant biomass in lignocellulose biorefineries [9,10].

The biotechnological interest of basidiomycetes has led to sequencing and analyzing a wide array of their genomes [11,12,13,14,15,16,17] with the purpose of better understanding global lignocellulose degradation processes and identifying enzymes that can be used as industrial biocatalysts. Most of these genomes have been sequenced at the US Department of Energy (DOE) Joint Genome Institute (JGI) (www.jgi.doe.gov; accessed on 25 April 2021) within a Fungal Genomics Program (2020 fungal genomes sequenced, including 594 basidiomycetes, updated to April 2021). Until recently, the sequenced basidiomycete genomes were mainly from species of the order Polyporales, which includes most wood rotting fungi. Their analysis revealed the complexity of the lignocellulolytic system of fungi with a variety of enzymes belonging to a multitude of oxidoreductase, hydrolase, esterase, and lyase families, among others. Comparative analysis of genomes, transcriptomes and secretomes provided evidence on the involvement of different oxidoreductases in lignin degradation, including peroxidases, laccases and the H_2_O_2_-generating oxidases necessary for peroxidase activity, and strongly supported the central role of peroxidases in ligninolysis by white-rot basidiomycetes [11,15,18,19,20,21,22,23,24,25]. In addition to the oxidoreductases identified so far, others are becoming available from the sequencing projects currently in progress. Thus, new lytic polysaccharide monooxygenases (LPMOs) active on cellulose-bound xylan, and novel oxidoreductase families that might be involved in lignin biodegradation in different environments and lignocellulosic substrates, have been recently identified from genome mining [17,26].

Lignocellulose-degrading *Pleurotus eryngii* is an edible mushroom and a grass-litter decomposer living as saprotrophic, or weakly parasitic, on the roots of herbaceous Apiaceae plants. Its mycelium is frequently grown on lignocellulosic wastes (such as wheat–straw), and fruit bodies are commercialized throughout the world, with Asian countries as the main producers (about 40,000 tons in Japan and 673,000 tons in China in 2013) [27]. Interestingly, it has been reported that *P. eryngii* is able to selectively degrade lignin when growing on non-woody lignocellulosic materials, becoming an organism of biotechnological interest for biopulping [28] and other applications where delignification is required, such as bioethanol production [29]. Given its applied interest, a variety of key enzymes involved in lignin degradation, including peroxidases, laccases, and aryl–alcohol oxidases, have been identified and characterized in this fungal species [30,31,32,33,34].

To improve our knowledge about the lignocellulolytic machinery of *P. eryngii*, we sequenced the genome of the monokaryotic strain ATCC 90797 [17]. Using this information, here we have addressed the study of this machinery during lignocellulose degradation using wheat–straw as substrate. We applied an integrated proteomic, transcriptomic and metabolomic approach with the aim of understanding how the fungal enzymes involved in plant cell-wall degradation are orchestrated.

## 2. Materials and Methods

### 2.1. Organism and Culture Conditions

Monokaryotic *P. eryngii* ATCC 90797, derived from heterokaryotic ATCC 36047 (isolated from Czechoslovakia), was obtained from the American Type Culture Collection (www.atcc.org; accessed on 25 April 2021). This fungus was grown in a medium containing (*w*/*v*) 2% malt extract, 2% glucose, 0.1% peptone and 2% agar, at 28 °C, and conserved at 4 °C.

Secretomic, transcriptomic, and chemical analyses were performed on glucose–ammonium and wheat–straw cultures of *P. eryngii*. The glucose–ammonium cultures were grown in 250-mL flasks with 50 mL of medium containing (*w*/*v*) 1% glucose, 0.2% ammonium tartrate, 0.1% KH_2_PO_4_, 0.05% MgSO_4_·7H_2_O, 0.05% KCl, 0.1% yeast extract and 1 mL/L trace elements solution, which consists of 100 mg/L Na_2_B_4_O_7_·10H_2_O, 10 mg/L CuSO_4_·5H_2_O, 50 mg/L FeSO_4_·7H_2_O, 10 mg/L MnSO_4_·4H_2_O, 10 mg/L (NH_4_)_6_Mo_7_O_24_·4H_2_O and 70 mg/L ZnSO_4_·7H_2_O. The lignocellulosic cultures were grown on 4 g of chopped wheat (*Triticum aestivum*) straw (particle size ~5–20 mm long × 1–3 mm wide) soaked with 10 mL of distilled water in 250-mL flasks (solid-state fermentation conditions). Inocula for both culture types consisted of mycelium from glucose–ammonium stationary cultures, which was homogenized, incubated for additional 15 days in the same medium, washed, resuspended in sterile distilled water and used for inoculation (4 mL/flask).

Both liquid and solid-state fermentation cultures were grown at 28 °C for 43 days under stationary conditions. Samples (entire flasks) were collected after 6, 14, and 43 days of incubation. Three replicates of the wheat–straw cultures were used for the total RNA extraction and lignocellulose composition analyses described in the next sections. Additional triplicates of the wheat–straw cultures were treated with 80 mL distilled water at 180 rpm and 24 °C for 100 min. These and the glucose–ammonium cultures were filtered under vacuum, and the filtrates were used for proteomic and aromatic-compound analyses, while the mycelium from glucose–ammonium cultures was used for total RNA extraction.

### 2.2. Library Preparation and RNA Sequencing

Total RNA used for transcriptomic analysis was isolated from 100 mg of (i) colonized wheat–straw, and (ii) mycelium from glucose–ammonium cultures. The samples were ground with FastPrep Lysing Matrix A (MP Biomedicals, Solon, OH, USA) in 1 mL of TRIzol reagent (Ambion, Austin, TX, USA). Nucleic acids were precipitated with isopropanol, resuspended in water and treated with DNase I (RNase-free, Qiagen, Hilden, Germany). Total RNA was precipitated with LiCl and resuspended in water treated with diethyl pyrocarbonate. RNA quantity and quality were determined using the Experion RNA StdSens kit (Qiagen). Double-stranded cDNAs were synthesized from Poly A RNA and fragmented (200–300 bp) before construction of the sequencing libraries (Kapa Library Amplification Kit; Kapa Biosystems, Wilmington, MA, USA). Eleven libraries were obtained corresponding to triplicates from wheat–straw and glucose–ammonium cultures at days 6 and 14 (except for the 14-day cultures on wheat–straw, with only two replicates since the third one did not pass the quality control thresholds using the Experion System).

Sequencing was done at JGI on an Illumina NovaSeq with 2 × 151 base pair (bp) reads. Raw fastq file reads were filtered and trimmed using the JGI QC pipeline. Filtered reads from each library were aligned to the reference genome of *P. eryngii* ATCC 90797 v1.0 using HISAT2 version 2.1.0 [35]. featureCounts [36] was used to generate the raw gene counts file. Only primary hits assigned to the reverse strand were included in the raw gene counts.

As a quality control we: (i) verified that the expression of 18 housekeeping genes remained constant and at the same levels each of them regardless of growth conditions and incubation time; (ii) measured the Pearson’s correlation between biological replicates; and (iii) performed a principal component analysis (PCA) of the expression patterns and tested statistically significant differences between conditions using PERMANOVA as implemented in the Adonis function of the R package vegan v.2.5–7 [37].

Genes with RNA-seq mean raw read counts superior to 5 were considered to be transcribed and selected for subsequent analysis. We used the DESeq2 Bioconductor package v.1.30.1 to normalize and transform to regularized log (rlog) the read counts of these genes. Then, we measured the differential expression patterns of *P. eryngii* grown on wheat–straw and in glucose–ammonium medium. Genes with log2(counts) ≥ 12 were considered highly transcribed (HT) and genes with a log2 fold-change (FC) ≥ 2 and Wald test false discovery rate (FDR) < 0.05 were considered up-regulated [38,39].

### 2.3. Protein Extraction and Detection

The replicate samples obtained for the proteomic analysis, as described above, were combined. Then, they were freeze-dried, resuspended in 20 mM sodium tartrate (pH 5.0), impurities removed by a short polyacrylamide gel electrophoresis run, and stained by colloidal Coomassie blue. The protein bands were cut, destained using 50 mM ammonium bicarbonate in 50% acetonitrile, and subjected to tryptic digestion [40]. Tryptic peptides were analyzed in an LTQ-Orbitrap Velos mass spectrometer coupled to an Easy-nLC 1000 HPLC system (Thermo Scientific, Waltham, MA, USA). Peptides were first loaded into an Acclaim PepMap 100 (Thermo Scientific) precolumn, and then eluted onto an Acclaim PepMap C18 column (25 cm long, 75 µm i.d., and 3 µm particle size) (Thermo Scientific) using a 120-min solvent gradient (solvent A: 0.1% formic acid in 2% acetonitrile; solvent B: 0.1% formic acid in pure acetonitrile) set as follows: 0–35% solvent B for 90 min, 35–45% solvent B for 10 min, 45–95% solvent B for 5 min, 95% solvent B for 10 min, 95–100% solvent B for 1 min, and 100% solvent B for 4 min, at a flow rate of 250 nL/min.

Mass spectrometry (MS) analysis was performed in the Orbitrap at 30,000 (at *m*/*z* 400) resolution using a 200–1600 *m*/*z* mass range. After the survey scan, the 15 most intense precursor ions were selected for CID (collision-induced dissociation) fragmentation in the ionic trap. Fragmentation was performed with a normalized collision energy of 35%. Charge state screening was enabled to reject unassigned and singly charged protonated ions. A dynamic exclusion time of 45 s was used to discriminate against previously selected ions.

MS data were analyzed with Proteome Discoverer (version 1.4.1.14) (Thermo Scientific) using standardized workflows. Acquired spectra were searched against the catalog of predicted proteins from the *P. eryngii* ATCC 90797 genome [17] (15,960 protein sequence entries) available from the JGI MycoCosm portal (https://mycocosm.jgi.doe.gov/Pleery1; accessed on 25 April 2021) using the SEQUEST search engine. Precursor and fragment mass tolerances were set to 10 ppm and 0.5 Da, respectively, and a maximum of two missed cleavages were allowed. Carbamidomethylation of cysteines was set as a fixed modification and methionine oxidation was set as a dynamic modification. Identified peptides were validated using the Percolator algorithm [41] with a q-value threshold of 0.01.

### 2.4. Automatic and Expert Gene Annotation

Gene functional annotation was initially based on the JGI automated annotation pipeline [42], which uses the following databases: Gene Ontology (GO), Kyoto Encyclopedia of Genes and Genomes (KEGG), InterPro (IPR), Eukaryotic Orthologous Groups (KOG) and Enzyme Commission numbers (EC number). Genes encoding carbohydrate-active enzymes, CAZymes [43], were annotated using this approach. Oxidoreductases directly or indirectly contributing to plant cell-wall degradation (classified as Auxiliary Activities, AAs, in CAZy database, http://www.cazy.org; accessed on 25 April 2021) were searched by BLASTing amino-acid sequences of reference enzymes against the catalog of predicted proteins from the *P. eryngii* genome as described by Ruiz-Dueñas et al. [17]. Then, they were functionally annotated based on: (i) alignment of their amino-acid sequences with those of reference enzymes whose functional properties are known; and (ii) identification of characteristic catalytic sites and/or structurally relevant residues in 3D-models generated at the automated protein structure homology-modeling server SWISS-MODEL [44]. Depending on their putative substrates, CAZymes and oxidoreductases (AAs) were catalogued as plant cell-wall degrading enzymes (PCWDEs) or fungal cell-wall degrading enzymes (FCWDEs). Proteins predicted to be secreted by the classical secretory pathway were identified using SECRETOOL [45]. Unconventionally secreted proteins were predicted using ApoplastP [46].

Transcriptomic data and exoproteomic information can be found in Table S1, which includes the automatic and expert gene functional annotations described above.

### 2.5. Gene Set Enrichment Analysis

Gene Ontology (GO) enrichment analysis of up-regulated genes identified as described in Section 2.2 was performed using the Bioconductor package topGO v.2.42.0 [47]. The chosen parameters were the “weight01” algorithm and a two-sided Fisher exact test with an FDR threshold of 0.01. We used REViGO [48] to remove redundant terms (SimRel < 0.5) and visualize the enrichment results. The three GO categories were analyzed: (i) Biological Process (BP); (ii) Molecular Function (MF); and (iii) Cellular Component (CC).

### 2.6. Targeted Homology Searches for Aromatic Catabolism

The amino-acid sequences of validated and putative enzymes for aromatics catabolism in *Trametes versicolor* and *Ceriporioposis subvermispora* [49] (Table S2) were BLASTed against the catalog of filtered predicted proteins from *P. eryngii* available at MycoCosm. We used the BLASTP algorithm through NCBI Standalone blast 2.10.0+ [50]. The number of hits was limited to two proteins for each query sequence. Any hits with e-value less than 1 × 10^−100^ and identity higher than 30% were selected for further comparative analyses.

### 2.7. Chemical Analyses

The straw samples treated and untreated with *P. eryngii*, as described above, were dried at 65 °C for a week, weighed, and milled (size < 0.2 mm). Solvent (acetone) extractives, hot-water-soluble material, acid-insoluble lignin (Klason lignin), acid-soluble lignin, and polysaccharide composition (from monosaccharides released by sulfuric acid hydrolysis) were determined by standard Tappi methods [51]. The analyses were carried out in triplicate.

Aromatic compounds and different carboxylic acids present in the extracellular medium were chromatographically analyzed. For that, extracts (20 mL) of wheat–straw in water, obtained as described above, were adjusted to pH 1.5 with HCl, and extracted three times with 15 mL of diethyl ether. Traces of water were removed with Na_2_SO_4_, the diethyl ether was evaporated overnight in a fume hood, and the extracted compounds were resuspended in 12.5 µL of pyridine (with 4 µg of ethylvanillin as the internal standard) and derivatized with 20 µL of bis(trimethylsilyl)-trifluoroacetamide at 50 °C for 10 min. The silylated compounds were analyzed by gas chromatography-mass spectrometry (GC–MS) on a 7980A GC/5975C MSD system (Agilent), using a DB5-HT column (30 m × 0.25 mm i.d. × 0.1 µm film thickness) programmed for the temperature to increase from 70 °C to 250 °C at 30 °C min^−1^. Then, they were identified by comparison of the mass spectra with the NIST library. Finally, the abundances were calculated by comparing their peak areas with that of the internal standard. The chromatographic analysis was carried out in triplicate, and means and 95% confidence limits were calculated.

Lipophilic compounds in the acetone extracts of the wheat–straw samples—including fatty acids, sterols, steroid ketones, fatty alcohols, steryl glycosides, waxes, and triglycerides—were studied by redissolving the dried acetone extract in chloroform, identified and quantified by GC with flame-ionization detection (using compounds representative of the above lipid classes for preparation of calibration curves). The GC–MS analyses were performed with a Varian Star 3400 gas chromatograph (Varian, Walnut Creek, CA, USA) coupled with an ion-trap detector (Varian Saturn 400) equipped with a high-temperature capillary column (DB-5HT, 15 m × 0.25 mm i.d., 0.1 μm film thickness; J&W Scientific, Santa Clara, CA, USA). Helium was used as carrier gas at a rate of 2 mL/min. The samples were injected with an autoinjector (Varian 8200) directly onto the column using a septum-equipped programmable injector (SPI) system. The temperature of the injector during the injection was 120 °C, and 0.1 min after injection was programmed to 380 °C at a rate of 200 °C min^−1^ and held for 10 min. The oven was heated from 120 °C (1 min) to 380 °C (5 min) at 10 °C min^−1^. The temperature of the transfer line was set at 300 °C. Bis(trimethylsilyl)trifluoroacetamide (BSTFA) silylation was used when required.

The wheat–straw treated with *P. eryngii* and the corresponding controls were analyzed by heteronuclear single quantum correlation (HSQC) ^13^C-^1^H NMR spectroscopy, using a Bruker Avance-III 500 MHz instrument equipped with a cryogenically cooled 5 mm TCI gradient probe with inverse geometry. In these experiments, the whole lignocellulosic samples were analyzed at the gel state in deuterated dimethylsulfoxide (DMSO-*d_6_*) as described previously [52]. The central peak of residual non-deuterated DMSO was used as the internal reference (at δ_C_/δ_H_ 39.5/2.49), and the spectra were normalized to the same intensity of the DMSO signals (same DMSO volume and amount of sample were used in all cases). The Bruker’s “hsqcetgpsisp.2” adiabatic pulse program was applied with spectral widths from 0 to 165 ppm (20,625 Hz) and from 0 to 10 ppm (5000 Hz) for the ^13^C and ^1^H dimensions, respectively. The ^1^*J*_CH_ used was 145 Hz. Signals were assigned by comparison with the literature [53].

## 3. Results

### 3.1. Repertoire of Plant Cell-Wall Degrading Enzymes in the Genome of P. eryngii

The genome of monokaryotic *P. eryngii* ATCC 90797 was sequenced in the frame of the JGI project “Study of the lignocellulolytic machinery in saprobic wood and leaf litter degrading Agaricales”. The sequencing process resulted in a 44.61-Mbp haploid genome assembled into 1684 contigs and 609 scaffolds (available at https://mycocosm.jgi.doe.gov/Pleery1/Pleery1.info.html; accessed on 25 April 2021). In total, 15,960 protein coding genes were predicted [17]. Out of these, 497 proteins (3% of the overall encoded proteins) constitute the repertoire of: (i) glycoside hydrolase (GH, 239 enzymes), polysaccharide lyase (PL, 33 enzymes) and carbohydrate esterase (CE, 26 enzymes) CAZymes; (ii) carbohydrate-binding modules (CBMs, 95 modules); and (iii) oxidoreductases classified as Auxiliary Activities (AAs, 104 enzymes, mainly including lytic polysaccharide monooxygenases, laccases, peroxidases and H_2_O_2_-generating oxidases) in CAZy database, among which the enzymes (and CBMs) directly or indirectly involved in the degradation of plant cell-wall polymers are found (Table S3). Besides these, unspecific peroxygenases (UPOs, 3 enzymes) and dye-decolorizing peroxidases (DyPs, 4 enzymes), which have been related to lignin degradation and transformation of lignin-derived compounds [7], were also identified in the *P. eryngii* genome (Table S3). Along with these enzymes, we identified 17 hydrophobins [54] (Table S3).

### 3.2. Proteins Mobilized by P. eryngii on Wheat–Straw and Glucose–Ammonium Cultures

**Figure 1:**
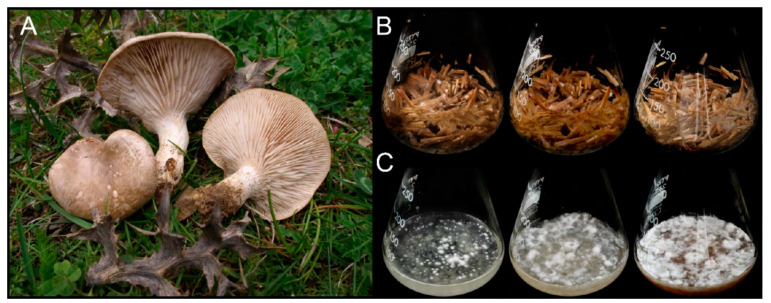
*Pleurotus eryngii* fruiting bodies (**A**), and monokaryotic (ATCC 90797) cultures on wheat–straw (**B**) and in glucose–ammonium medium (**C**) after 6 (**left**), 14 (**center**) and 43 (**right**) days of culture (image of fruit bodies courtesy of Dr J.M. Barrasa).

To understand how the enzymatic machinery responsible for plant cell-wall degradation is orchestrated by *P. eryngii* (Figure 1A), we grew this fungus on wheat (*T. aestivum*) straw (with distilled water as the only additive) under the so-called solid-state fermentation conditions for 43 days (Figure 1B), a period of time considered a priori to be long enough to achieve a complete metabolic adaptation to this substrate. Gene expression was evaluated by RNA-seq of samples obtained after 6 and 14 days of culture, and the exoproteome (proteins released to the extracellular medium) was analyzed by nLC-MS/MS of the total peptides resulting from trypsin hydrolysis of samples collected on days 6, 14, and 43. As described below, transcript levels, and diversity and relative abundance of the identified proteins were compared to those found in cultures of *P. eryngii* grown in the absence of lignocellulosic substrate in glucose–ammonium medium (Figure 1C).

A preliminary analysis of the global transcriptomic response of *P. eryngii* to the culture conditions revealed a total of 11,845 transcribed genes (74% of all genes in the genome). Surprisingly, transcripts for 97% of the CAZymes and AA oxidoreductases (and 87% of CBMs) encoded in the genome were detected when growing either on wheat–straw or in glucose–ammonium medium.

**Figure 2:**
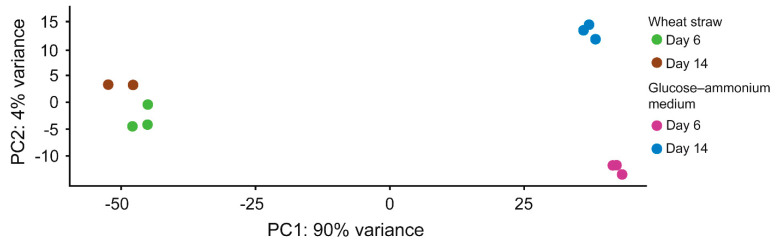
Principal component analysis of the log2 normalized RNA-seq read counts of 11 biological replicates of *P. eryngii* growing on wheat–straw and in glucose–ammonium medium for 6 and 14 days. The replicates are shown as colored circles according to the growth conditions: (i) green and brown for cultures of 6 and 14 days on wheat–straw, respectively; and purple and blue for 6 and 14 days of culture in glucose–ammonium medium, respectively.

To determine the consistency of the RNA-seq quantitative data, a total of 18 housekeeping genes were analyzed. They showed a stable transcript level regardless of growth conditions and incubation time (Figure S1), confirming that all our biological conditions were comparable to each other. Then, a Pearson’s correlation matrix (Figure S2) and a principal component analysis (PCA) of the log2 normalized RNA-seq read counts from the biological replicates (Figure 2) confirmed statistically significant differences between the expression patterns in both culture media (PERMANOVA *p*-value = 0.0025). These differences were assumed to be the result of the adaptation of *P. eryngii* to grow on wheat–straw.

**Figure 3:**
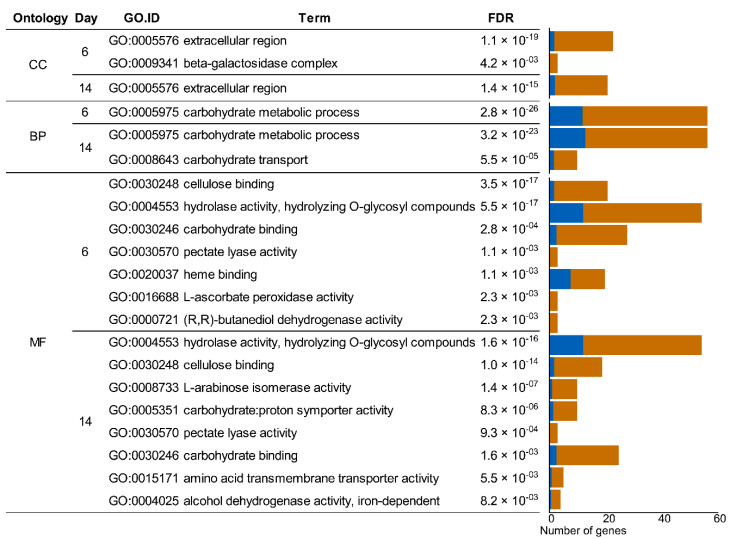
Enrichment of up-regulated genes in *P. eryngii* when growing on wheat–straw compared with glucose–ammonium medium, clustered by the GO categories: CC, “Cellular Component”; BP, “Biological Process”; and MF “Molecular Function”. The bar chart shows the expected (blue) and observed (brown) number of up-regulated genes of each GO term.

The biological terms showing a higher proportion among up-regulated genes (log2 FC ≥ 2 and FDR < 0.05) than the proportion of genes in the genome were identified using Gene Ontology (GO) enrichment analysis (Figure 3). Thus, extracellular proteins (GO: 0005576) involved in carbohydrate metabolism (GO: 0005975), and more specifically enzymes implicated in carbohydrate-binding (GO:0030248 and GO:0030246) and hydrolysis (including cellulose, hemicellulose and pectin) (GO:0004553 and GO:0030570), appeared enriched in wheat–straw on day 6 and 14 of growth. Heme proteins, including ligninolytic class-II peroxidases (AA2, POD) and cytochrome P450 monooxygenases (P450s) (GO:0016688 and GO:0020037), were overrepresented in the transcriptome on day 6, while proteins involved in carbohydrate and amino-acid transport (GO:0005351 and GO:0015171) were enriched on day 14 (for more details see Table S4).

### 3.3. Diversity and Abundance of Main Enzyme Types in the Exoproteome of P. eryngii

**Figure 4:**
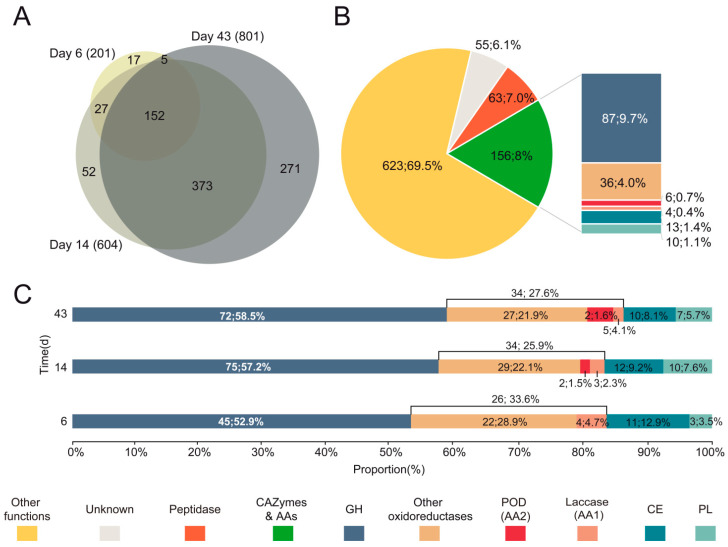
Functional distribution of proteins identified in the exoproteome of *P. eryngii* grown on wheat–straw. (**A**) Venn diagram of total protein numbers (897) after 6 (blue), 14 (red) and 43 (green) days of culture. (**B**) Distribution into functional groups. (**C**) Distribution of CAZymes and oxidoreductases on 6, 14, and 43 days of culture. The number of proteins is followed by their percentage in (**B**,**C**), and their predicted functions are indicated by color codes.

Differences were also observed at exoproteome level, with 897 extracellular proteins identified in wheat–straw (Figure 4A), representing a 4.75-fold higher number than found (189 proteins) in cultures containing glucose and ammonium, as easily assimilated C and N sources (Figure S3A). The number of proteins increased over time, from 201 to 604 and 801 in wheat–straw (Figure 4A) and from 28 to 96 and 142 in glucose–ammonium medium at days 6, 14, and 43, respectively (Figure S3A). Peptidases playing an important role in the recycling of extracellular organic nitrogen, CAZymes, AAs, and proteins of unknown function represent a lower percentage in wheat–straw, compared to glucose–ammonium medium, due to the large number of proteins with “other functions” released to the extracellular space (Figure 4B and Figure S3B). However, their total content was higher in wheat–straw throughout the 43 days of culture, with 55 vs. 24 unknown proteins, 63 vs. 24 peptidases, and 156 vs. 85 CAZymes plus oxidoreductases (AAs). An adaptive response of *P. eryngii* to wheat–straw, described in more detail below, was observed at day 6, with the secretion of 59 CAZymes (45 GHs, 11 CEs and 3 PLs) and 26 AA oxidoreductases (Figure 4C), compared to its growth in glucose–ammonium medium where only 3 GHs and 6 AA oxidoreductases were detected (Figure S3C). Interestingly, following this initial period, the overall composition of the CAZymes and AAs set increased in protein number, and then was maintained relatively stable over time regarding the proportion of GHs (57–58%), CEs (8–9%), PLs (5–7%) and AAs (26–27%) on wheat–straw (Figure 4C), unlike what observed in glucose–ammonium medium where, on the other hand, CEs, and PLs were not identified until day 14 and 43, respectively (Figure S3C).

**Figure 5:**
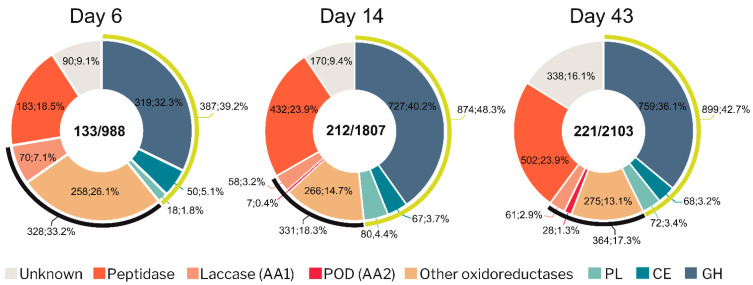
Abundance in PSM values (followed by the percentage) of the main protein types, indicated by color codes, in the exoproteome on wheat–straw cultures after 6, 14, and 43 days of incubation. The central numbers correspond to the number of secreted proteins (left) and their abundance in PSM values (right). Black and yellow arcs indicate oxidoreductases and CAZymes, respectively.

A semi-quantitative analysis of the exoproteomes was performed based on the peptide-spectrum match (PSM) values of each of the proteins identified by at least two unique peptides. Discarding proteins with a priori “other functions” not related to the degradation of the plant cell-wall (Figure 4B), a greater abundance of the rest of the proteins was detected in wheat–straw (including CAZymes, oxidoreductases and also peptidases and proteins with unknown function) compared to the glucose–ammonium medium (988 vs. 66 PSM, 1807 vs. 448 PSM and 2103 vs. 1209 PSM at day 6, 14, and 43, respectively) (Figure 5 and Figure S4). Noteworthy differences were found at day 6, with a high abundance of oxidoreductases and CAZymes (including GHs, CEs and PLs) identified in wheat–straw as a consequence of the adaptation of the fungus to this lignocellulosic substrate (Figure 5 vs. Figure S4). The relative abundance of oxidoreductases was reduced in wheat–straw from this day due to the increase of CAZymes. Within the oxidoreductase group, laccases presented a similar relative abundance in wheat–straw and glucose–ammonium medium (except for day 6). Interestingly, although PODs represent a low percentage of the proteins in the exoproteome since day 14, these enzymes increased their abundance in wheat–straw from this day to day 43 and were absent in the glucose–ammonium medium during the whole incubation time, supporting their role in lignocellulose degradation. Although with a low abundance, CEs and PLs were present in wheat–straw from day 6 (Figure 5) unlike what happened in glucose–ammonium medium, where CEs were not identified until day 14, also at low abundance, and the amount of PLs was negligible at day 43 (Figure S4), suggesting a relevant role of these enzymes in plant biomass transformation by *P. eryngii*.

### 3.4. Analysis of the Enzymatic Arsenal Activated by P. eryngii Growing on Wheat–Straw

To analyze in detail how the changes at the transcriptomic and proteomic levels contributed to the lignocellulolytic capabilities of *P. eryngii*, we merged the PSM values of the proteins identified in the exoproteome with the normalized read counts of the 11,845 transcribed genes, on both wheat–straw and glucose–ammonium medium (Table S1). The genes expressed on wheat–straw were classified as: (i) highly transcribed (HT), with a log2 normalized read counts value ≥ 12; (ii) up-regulated, with a log2-fold-change mean value ≥ 2 (and Wald test FDR < 0.05) compared to the expression in glucose–ammonium medium; and (iii) basally expressed, with a log2 normalized read counts value < 12 and a log2-fold-change mean value < 2.

**Figure 6:**
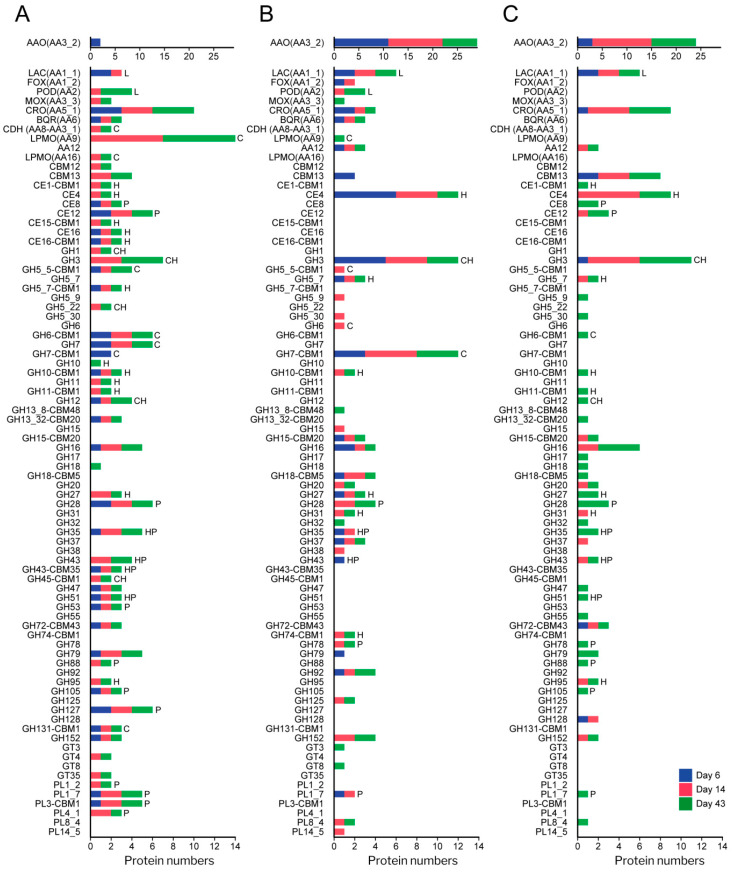
Diversity of CAZymes and oxidoreductases (AA) in the exoproteome of *P. eryngii* grown on wheat–straw and in glucose–ammonium medium. (**A**) Number of up-regulated (from Figure 7) and a few only highly transcribed (from Table S5), and (**B**) basally expressed (from Figure 8) enzymes of each (sub)family identified in the exoproteome of wheat–straw; and (**C**) number of enzymes identified in glucose–ammonium medium. The length of the bars represents the total number (sum) of proteins of each CAZyme and AA (sub)family identified on day 6 (blue section), 14 (red section) and 43 (green section). Although transcriptomic data were not available for day 43, we assumed that regulation of day 14 was maintained considering that the proportion of GHs, CEs, PLs and AAs was relatively stable (Figure 4C). The substrates on which the enzymes act are indicated: C, cellulose; H, hemicellulose; L, lignin; and P, pectin. Although laccases (Lac) and PODs are the only oxidoreductases here assigned a function (acting on lignin); AAO, methanol oxidases (MOX) and CRO (producing H_2_O_2_); and benzoquinone reductases (BQR) can be considered to be indirectly involved in lignocellulose degradation.

**Figure 7:**
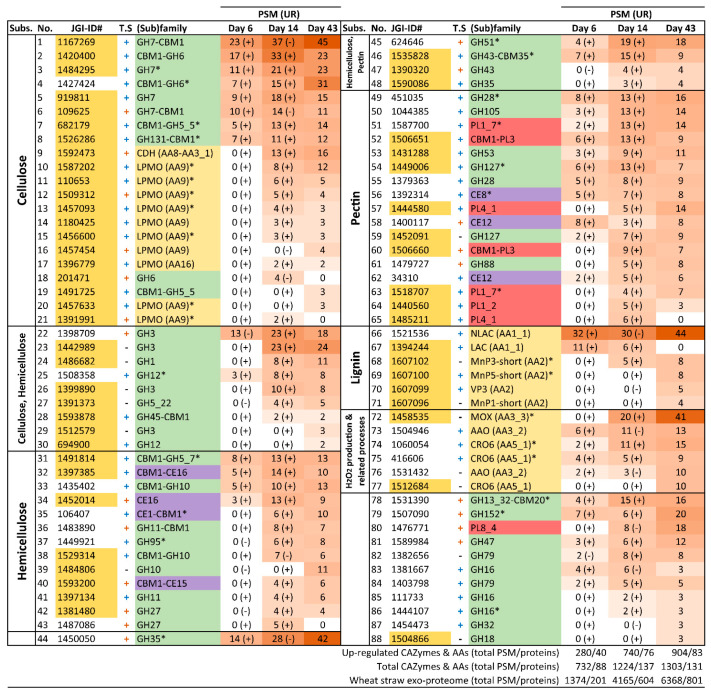
Up-regulated CAZymes and oxidoreductases (AA) identified in the wheat–straw exoproteome. The enzymes are organized according to the substrate on which they act or the process in which they participate (column “Subs”) and then sorted from highest to lowest abundance based on total PSM values. The protein reference numbers (JGI-ID#) correspond to those in the catalog of predicted proteins from the *P. eryngii* ATCC 90797 genome available through JGI MycoCosm Genome Portal. Protein references over yellow background are enzymes exclusively secreted on wheat–straw. The presence (+) or absence (-) of theoretical secretion (T.S) is indicated. Symbol + in blue or orange color represents proteins predicted to be secreted by the classical secretory pathway or by the unconventional pathway, respectively. Presence (+) or absence (-) of up-regulation (UR) in wheat–straw is indicated at day 6 and 14 following the PSM values. Enzymes 78–88 have not been related to lignocellulose degradation. * Proteins found both up-regulated and highly transcribed. Colors in the (sub)family column correspond to: GH, green; oxidoreductases, yellow; PL, red; and CE, purple.

**Figure 8:**
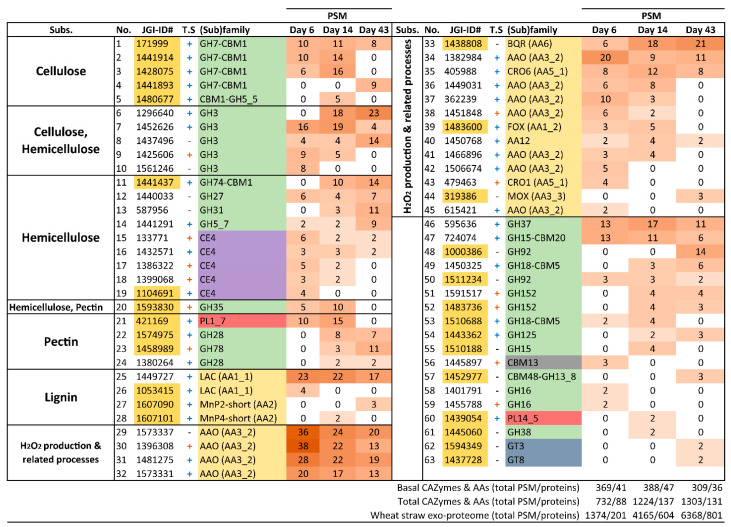
CAZymes and oxidoreductases (AA) transcribed at basal level identified in the wheat–straw exoproteome. The enzymes are organized according to the substrate on which they act or the process in which they participate (column “Subs”) and then sorted from highest to lowest abundance based on total PSM values. The protein reference numbers (JGI-ID#) correspond to those in the catalog of predicted proteins from the *P. eryngii* ATCC 90797 genome available through JGI MycoCosm Genome Portal. Protein references over yellow background correspond to enzymes exclusively secreted on wheat–straw. The presence (+) or absence (-) of theoretical secretion (T.S) is indicated. Symbol + in blue or orange color represents proteins predicted to be secreted by the classical secretory pathway or by the unconventional pathway, respectively. Enzymes 46–63 have not been related to lignocellulose degradation. Colors in the (sub)family column correspond to: GH, green; oxidoreductases, yellow; PL, red; CE, purple; GT (glycosyl transferase), blue; and CBM, gray.

The number of CAZyme and oxidoreductase (AA) families represented in the exoproteome of the glucose–ammonium cultures throughout the 43 days of incubation (a total of 42, including 4 AA, 4 CE, 32 GH and 2 PL families) (Figure 6C) increased by 64% when *P. eryngii* was grown on wheat–straw (a total of 65, including 11 AA, 6 CE, 42 GH and 6 PL families) with both up-regulated and basally expressed enzymes (Figure 6A,B). Out of these, at least one member of each of the families directly or indirectly acting on the plant cell-wall appeared up-regulated (and a few HT) (a total of 41, including 9 AA, 6 CE, 22 GH, and 4 PL families) (Figure 6A and Figure 7; and Table S5 for detailed enzyme information). GH31, GH74, and GH78 were the exception, since they were only represented by enzymes transcribed at basal levels (Figure 6B). On the other hand, most of the CAZymes and oxidoreductases (AA) identified in the wheat–straw exoproteome were exclusively secreted on this lignocellulosic substrate (enzymes with their JGI-IDs over yellow background in Figure 7 and Figure 8, and Table S5) since they were absent from the glucose–ammonium exoproteome. Most of the enzymes identified in wheat–straw (131 of 164) were predicted to be secreted proteins that move through the secretory pathway (92 proteins, as determined with SECRETOOL) or are unconventionally secreted independently of a signal peptide (39 proteins according to ApoplastP) (column T.S in Figure 7 and Figure 8, and Table S5). The presence in the exoproteome of enzymes whose secretion could not be predicted by SECRETOOL or ApoplastP suggests the existence of different alternative secretion mechanisms although their release by cell lysis cannot be ruled out.

#### 3.4.1. Polysaccharide-Decay Machinery

**Figure 9:**
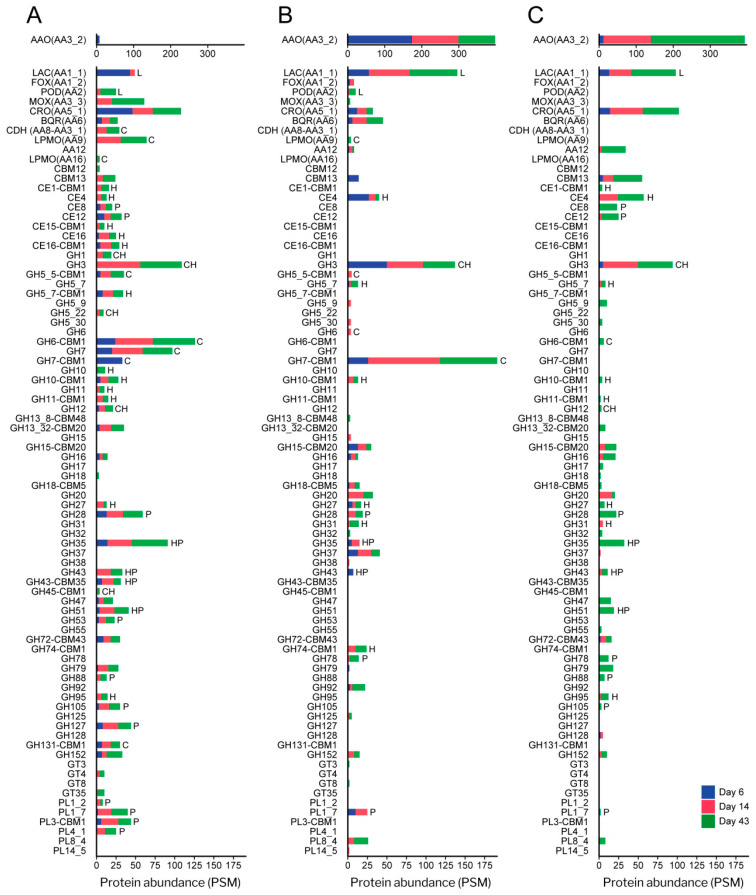
Abundance (in PSM values) of CAZymes and oxidoreductases (AA) in the exoproteome of *P. eryngii* grown on wheat–straw and in glucose–ammonium medium. (**A**) Abundance of up-regulated (from Figure 7) and a few ones only highly transcribed (from Table S5), and (**B**) basally expressed (from Figure 8) enzymes of each (sub)family identified in the exoproteome on wheat–straw, and (**C**) abundance of enzymes identified in glucose–ammonium medium. The length of the bars represents the total abundance (sum) of proteins of each CAZyme and AA (sub)family identified on day 6 (blue section), 14 (red section) and 43 (green section). Given that transcriptomic data were not available for day 43, we assumed that regulation of day 14 was maintained also on day 43 considering that the overall composition of CAZymes and AAs was relatively stable over time regarding the proportion of GHs, CEs, PLs and AAs on wheat–straw (Figure 4C). The substrates on which the enzymes act are indicated: C, cellulose; H, hemicellulose; L, lignin; and P, pectin. Although laccases (Lac) and PODs are the only oxidoreductases here assigned a function (acting on lignin); AAO, MOX and CRO (producing H_2_O_2_); and BQR can be considered indirectly involved in lignocellulose degradation.

A wide range of glycoside–hydrolases involved in cellulose depolymerization (appended and non-appended to a CBM1 domain) were identified in the wheat–straw exoproteome on day 6 (Figure 7 and Figure 8, and Table S5), including 11 endo- and exo-β-1,4-glucanases (seven GH7, two GH6, one GH5_5 and one GH12 enzymes), five GH3 β-glucosidases and one enzyme of the multifunctional family GH131 described to be active on β-1,4, β-1,3 and mixed β-1,4/1,3 glucosidic linkages [55]. Most of the above enzymes were up-regulated in wheat–straw from day 6 (Figure 6A and Figure 7), with all members of GH3 family and some representatives of GH7 family expressed at basal transcription levels (Figure 6B, Figure 7 and Figure 8), the latter yielding amounts of extracellular proteins similar to those resulting from up-regulated genes (Figure 9). Unlike observed in wheat–straw, GHs active on cellulose were restricted to only a member of the GH3 family in glucose–ammonium medium on day 6. The GH6, GH7 and GH3 enzymes were the most secreted CAZymes on wheat–straw not only on day 6 but during the 43 days of culture (Figure 9A,B) confirming the key role assigned to these enzymes in cellulose depolymerization. Over time (after 14 and 43 days of culture), the repertoire of glycoside–hydrolases made up of all the above enzymes putatively improved the cellulolytic capabilities of *P. eryngii* on wheat–straw with the increase in the number of GH3, GH6 and GH12 enzymes, and the appearance of representatives of new GH families in the exoproteome, including one GH1 β-glucosidase, one GH45 and one GH5_22 enzymes, the two latter with putative endo-β-1,4-glucanase activity, all these enzymes being up-regulated from day 6 and 14, respectively (Figure 7).

Different oxidoreductases were also identified on wheat–straw completing the enzyme set responsible for cellulose cleavage. Among them, AA9 LPMO (active on crystalline cellulose) was the cellulolytic family with the highest number of representatives (nine enzymes) in the exoproteome (Figure 7). Every single AA9 LPMO, the only AA16 LPMO (also active on crystalline cellulose) and cellobiose dehydrogenase (CDH, oxidizing cellobiose and cellooligosaccharides to lactones, reducing aromatic radicals, and activating LPMOs) were up-regulated on day 6. However, it was not until day 14 that these oxidoreductases were identified in the exoproteome (Figure 6A and Figure 7). Then, the LPMO and CDH enzyme composition remained relatively stable until day 43, whereas these oxidoreductases were completely absent in the glucose–ammonium medium during the 43 days of culture (Figure 6C).

Compared with cellulose, hemicelluloses depolymerization requires a greater diversity of enzymes given the variety and heterogeneity of these polymers. Taking this into account, a fairly limited set of hydrolases active on hemicelluloses was deployed by *P. eryngii* on wheat–straw on day 6, including one up-regulated GH10 endo-β-1,4-xylanase and two GH5_7 endo-β-1,4-mannanases (one of them as an up-regulated and highly transcribed enzyme) able to cleave the xylan and mannan backbone, respectively (Figure 6, Figure 7, Figure 8 and Figure 9). Moreover, the GH12 and GH3 enzymes, putatively involved in cellulose degradation, could also act as xyloglucan endo-β-1,4-glucanase and β-1,4-xylosidase, respectively, releasing xylose units according to the activities described for these two GH families. These hydrolases were accompanied in the exoproteome by debranching enzymes able to remove side chains linked to the hemicellulose backbone, including GHs from families 27 (α-galactosidase), 35 (β-galactosidase), 51 (α-l-arabinofuranosidase), 43 (with a wide variety of described activities for this family, such as L-arabinofuranosidase, endo-α-l-arabinanase and β-d-xylosidase), and carbohydrate–esterases from families 4 and 16 (Figure 7 and Figure 8, and Table S5).

The composition of the enzyme set active on hemicelluloses evolved over time with the incorporation of new GHs and CEs to the already existing in the exoproteome on day 6, most of them up-regulated. Among others, further endoxylanases were identified on days 14 and 43. These included two more GH10 and two GH11 enzymes (both up-regulated) (Figure 7), the latter being more efficient on insoluble substrates, due to their smaller size, although they do not tolerate high substitutions on the xylan backbone. Along with these, new debranching enzymes appeared from families GH27 (α-galactosidase), GH35 (β-galactosidase), GH95 (α-fucosidase), GH5_22, GH43, CE1 (acetylxylan esterase according to Ruiz-Dueñas et al. [17]) and CE15 (4-O-methyl-glucuronoyl methylesterase) that cleaves the ester bond between lignin and glucuronoxylan [56]) (Figure 7).

Concerning the machinery involved in pectin depolymerization, there was a great variety of enzymes in the wheat–straw exoproteome from day 6 (Figure 7 and Figure 8). Among them, at least one representative of the following families appeared up-regulated this day in the transcriptome: (i) GH28 (which includes exo- and endo-polygalacturonases, exo- and endo-rhamnogalacturonases and xylogalacturan hydrolases), PL1_7 and PL3 pectate lyases, and GH105 unsaturated rhamnogalacturonyl hydrolase, contributing to break down the pectin backbone; and (ii) GH53 endo-β-1,4-galactanase, GH127, CE8 pectin methylesterase, and CE12 pectin acetylesterase acting as debranching enzymes. The pectinolytic machinery was completed with up-regulated members from families GH35 and GH51, also described as putatively involved in hemicellulose depolymerization; in addition to basally expressed enzymes from PL1_7 pectate lyase and GH35 families. The subsequent evolution of the pectinolytic machinery basically consisted of the extracellular accumulation of additional enzymes, thus improving the fungal pectin depolymerization capabilities. Further new enzymes on days 14 and 43 were up-regulated PL1_2 pectate lyases, PL4_1 rhamnogalacturonan endolyase, and GH88 unsaturated glucuronyl hydrolase (Figure 7), in addition to basally expressed GH78 α-rhamnosidase and GH28 enzymes (Figure 8).

#### 3.4.2. CBM-Containing Plant Cell-Wall Degrading Enzymes (PCWDEs)

**Figure 10:**
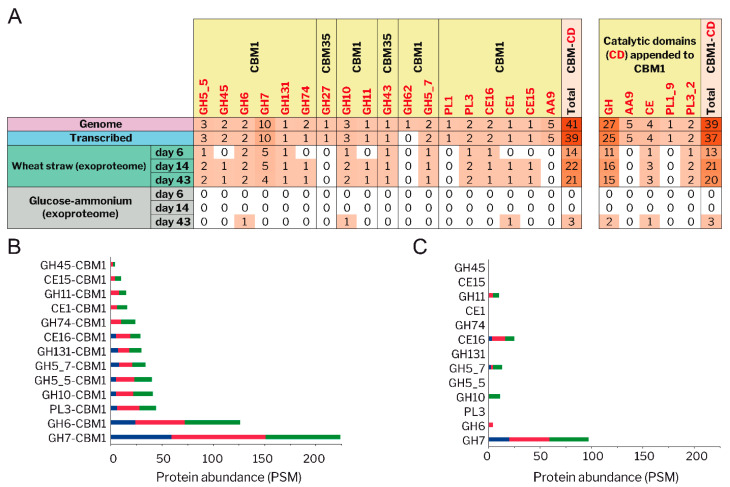
CBM-containing PCWDEs: (**A**) Catalytic domains (GHs, PLs, CEs and AA9 LPMOs) appended to CBM1 and CBM35 modules in the *P. eryngii* genome, transcriptome, and exoproteomes from wheat–straw*,* and glucose–ammonium cultures. (**B**,**C**) Abundances of catalytic domains appended and non-appended to CBM1, respectively, in the wheat–straw exoproteome after 6 (blue), 14 (red) and 43 (green) days of culture.

An analysis of the PCWDEs encoded in the genome of *P. eryngii* revealed that 41 enzymes are appended to carbohydrate-binding modules able to bind plant cell-wall polymers, and that most of them (39 of 41) are CBM1-containing PCWDEs which bind to crystalline cellulose (Figure 10A). The 41 CBM-appended enzymes include five AA9 LPMOs and members of 12 glycoside hydrolase (sub)families (GH5_5, GH5_7, GH6, GH7, GH10, GH11, GH27, GH43, GH45, GH62, GH74 and GH131), three carbohydrate–esterases (CE1, CE15 and CE16), and two polysaccharide–lyases (PL1_9 and PL3). A large number of these enzymes are released to the extracellular medium by *P. eryngii* when growing on wheat–straw (14, 22 and 21 CBM-containing PCWDEs after 6, 14, and 43 days respectively), unlike what observed when it was grown in glucose–ammonium medium, with only 3 CBM-containing PCWDEs secreted on day 43 (Figure 10A). Interestingly, when growing on wheat–straw, *P. eryngii* showed a preference for secreting CBM1 containing enzymes of those PCWDE families that have representatives both appended and non-appended to CBM1 (Figure 10B,C).

#### 3.4.3. Lignin-Decay Machinery

**Figure 11:**
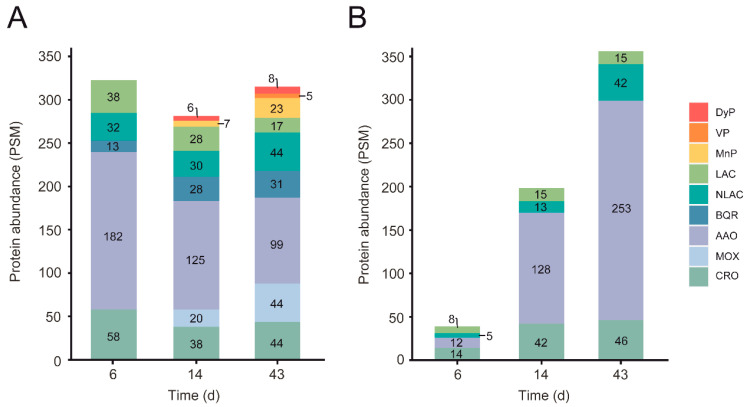
Abundance of main lignin-active oxidoreductases in the wheat–straw (**A**) and glucose–ammonium (**B**) exoproteomes of days 6, 14, and 43. Enzyme families: AAO, aryl–alcohol oxidases; BQR, benzoquinone reductases; CRO, copper-radical oxidases; DyP, dye-decolorizing peroxidases; LAC, laccases; MnP, manganese peroxidases; MOX, methanol oxidases; NLAC, novel laccases; VP, versatile peroxidases.

Unlike depolymerization of cell-wall polysaccharides—in which hydrolases, lyases, esterases, and oxidoreductases are involved—lignin degradation is basically an oxidative process. Among the oxidoreductases produced by *P. eryngii* potentially contributing to this process, three families were identified in the exoproteomes of both wheat–straw and glucose–ammonium cultures throughout the 43 days of incubation. The JGI-IDs and abundances of these enzymes are given in Figure 7 and Figure 8, Table S5, and in Figure 11, respectively, including: (i) phenol-oxidizing laccases of the multi-copper oxidase (MCO) superfamily, corresponding to the laccase sensu stricto (LAC) and novel laccase (NLAC) subfamilies [17]; (ii) aryl–alcohol oxidases (AAO) of the glucose-methanol-choline oxidase/dehydrogenase (GMC) superfamily, as the most abundant extracellular proteins identified; and (iii) copper-radical oxidases (CRO). The two latter enzymes are able to produce H_2_O_2_, which generate an extracellular oxidative environment and has been described to play different roles in lignocellulose degradation [24,57].

The main differences between the wheat–straw and glucose–ammonium cultures concern both the abundance of the secreted oxidoreductases, which was higher in wheat–straw, especially the first days; and the composition of the enzymatic set, with a few enzymes secreted specifically on wheat–straw (Figure 11). These enzymes included: (i) benzoquinone reductases (BQR) (CAZy family AA6), with a putative role in detoxifying quinones from lignin catabolism [58] and providing hydroquinone substrates for LPMO activation [59], present in the exoproteome from day 6 to day 43; (ii) methanol oxidases (MOX) also of the GMC superfamily (but with a substrate specificity different to that of AAOs) increasing the diversity of the pool of H_2_O_2_-producing enzymes from day 14; (iii) PODs of the manganese peroxidase (MnP) family from day 14 and versatile peroxidase (VP) family on day 43, both enzymes being able to oxidize lignin using Mn^3+^ as mediator, or directly in the case of VPs; and (iv) a member of the DyP family (JGI-ID 1429886), a peroxidase family that is not classified as AA in CAZy database although it has been described to contribute to the degradation of lignin-derived products.

Among the above oxidoreductases, the only NLAC enzyme (JGI-ID 1521536) appeared up-regulated on wheat–straw at day 6 (as also observed for LAC JGI-ID 1394244). This was among the most abundant enzymes in the exoproteome during the 43 days of culture (Figure 7) together with LAC JGI-ID 1449727, which was transcribed at basal levels (Figure 8). Although two AAOs were also up-regulated on wheat–straw (Figure 7), these two specific enzymes were poorly represented in the exoproteome (Figure 9A) compared with the remaining 11 secreted AAOs that, transcribed at basal levels (Figure 9B), were the main oxidoreductases in five of the six exoproteomes analyzed (Figure 11). Unlike AAOs, the most abundant CRO enzyme was a CRO2 (JGI-ID 1511425) highly transcribed both on day 6 and day 14 (Table S5). Concerning PODs, three MnP (MnP1, MnP3 and MnP5 with JGI-IDs 1607096, 1607102, and 1607100, respectively) and two VP (VP1 and VP3 with JGI-IDs 1607098 and 1607099, respectively) genes were up-regulated on day-6 transcriptome (Figure 7 and Table S5). However, it was not until day 14 that we found two PODs in the exoproteome (Figure 7 and Figure 8), namely MnP3 and MnP4 with the latter transcribed at basal levels. Then, the abundance of PODs continued to increase on day 43 with four MnPs (MnP1, MnP3, MnP4 and MnP5) and one VP (VP3) (Figure 7 and Figure 8).

### 3.5. Catabolism of Simple Aromatic Compounds

Intracellular pathways for the catabolism of lignin-derived aromatic compounds have recently been described in the white-rot basidiomycetes *T. versicolor* and *C. subvermispora* [49]. Using proteins from these pathways as queries, we performed BLASTp homology searches against the catalog of predicted proteins from the *P. eryngii* genome. As a result, a total of 32 genes encoding enzymes putatively involved in the conversion of 4-hydroxybenzoic acid (pHBA, exhibiting equivalent ring functionality to the *p*-hydroxyphenyl motif in lignin, and the *p*-coumaric acid present in wheat–straw as described in Section 3.6) were identified (Table S2). Some of them are up-regulated and/or highly transcribed in wheat–straw, and a few were also identified in the exoproteome (Table S6) despite not presenting secretion signal peptide. This is most likely due to the high expression levels observed in these cultures, and a consequence of hyphal lysis.

**Figure 12:**
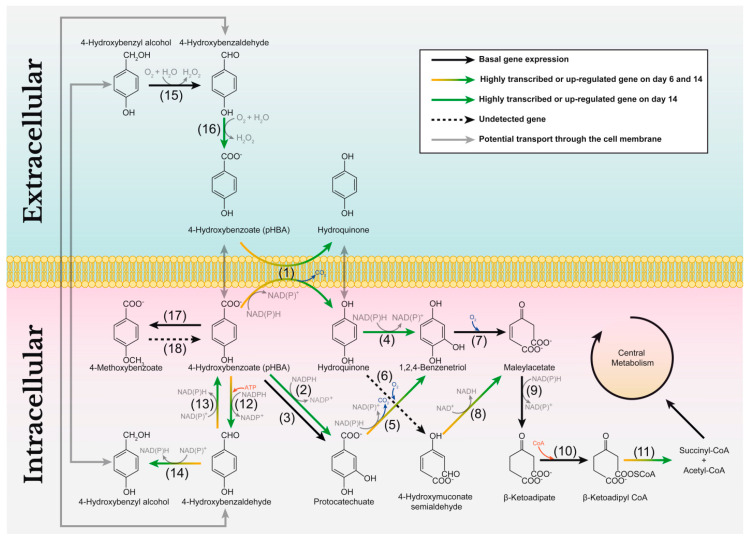
Proposed pathways for the conversion of pHBA by *P. eryngii*. Continuous arrows indicate enzymatic steps for which enzymes were found in the genome. The color codes indicate at least one of these enzymes expressed (from RNA-seq data) as described in the inset. Proposed enzymatic steps: (1) oxidative decarboxylase, (2) hydroxylase, (3) hydroxylation by P450s, (4) hydroxylase, (5) oxidative decarboxylase, (6) dioxygenase, (7) ring-cleaving dioxygenase, (8) 4-hydroxymuconic semialdehyde dehydrogenase, (9) maleylacetate reductase, (10) ketoacid-CoA transferase, (11) thiolase, (12) carboxylic acid reductase, (13) aldehyde dehydrogenase, (14) alcohol dehydrogenase, (15) alcohol oxidase, (16) aldehyde oxidase, (17) 4-*O*-methyl transferase and (18) demethylase (see Table S6 for additional information).

The above 32 enzymes cover 18 enzymatic steps in the pHBA catabolism (Figure 12 and Table S6). Thus, we hypothesize that once pHBA enters the cell, it may be converted to hydroquinone and protocatechuate by up-regulated oxidative decarboxylase (JGI-ID 1521705) and hydroxylase (JGI-ID 1471020) enzymes (Figure 12, enzymatic steps 1 and 2), respectively, or related enzymes. The versatility of these enzymes could also lead the transformation into 1,2,4-benzenetriol (enzymatic steps 4 and 5) that would be cleaved into maleylacetate by a dioxygenase (JGI-ID 1440135) with basal gene expression (enzymatic step 7). Then, maleylacetate reduction via a basally expressed reductase would generate β-ketoadipate (enzymatic step 9). Finally, two further steps could convert β-ketoadipate into β-ketoadipyl-CoA via ketoacid-CoA transferase (enzymatic step 10), and highly transcribed thiolases would generate metabolites (succinyl-CoA and acetyl-CoA) entering multiple pathways (enzymatic step 11) including the tricarboxylic acid cycle and the fatty-acid biosynthesis.

Enzymes for alternative pHBA catabolic steps were also identified. Four putative carboxylic acid reductases (one of them up-regulated, JGI-ID 1431563) and eight putative alcohol dehydrogenases (four of them up-regulated) (Table S6) could catalyze successively the transformation of 4-hydroxybenzoate into 4-hydroxybenzaldehyde and then into 4-hydroxybenzy alcohol (Figure 12, enzymatic steps 13 and 14). This compound, once it is in the extracellular space, could be used as a substrate for H_2_O_2_ generation by one of the two alcohol oxidases identified in the BLASTp homology search (Table S6) or by an AAO (enzymatic step 15) as suggested by Guillén et al. [60].

### 3.6. Degradation of Wheat–Straw Components

**Figure 13:**
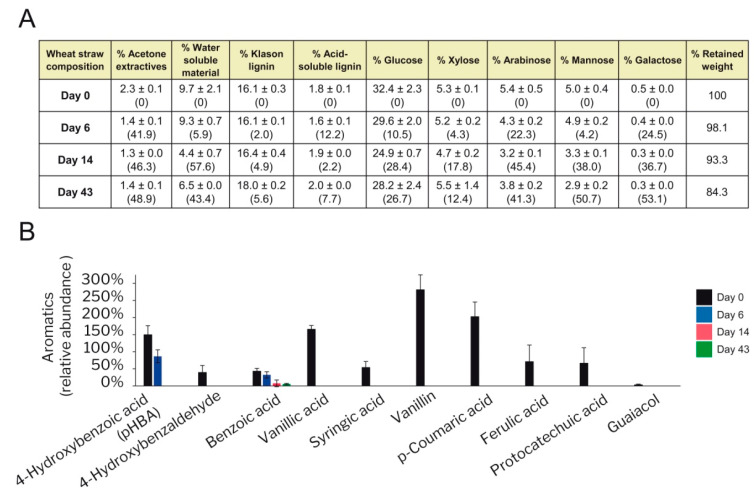
Degradation of wheat–straw components by *P. eryngii* over time. Relative abundance of: (**A**) major lignocellulose fractions (% of sample dry weight, with the degradation percentage with respect to day-0 in parentheses); and (**B**) polar aromatic compounds (vs ethylvanillin used as the internal standard at a concentration of 4 µg/g sample) in the water fraction before inoculation (Day 0, black columns) and after 6 (blue), 14 (red) and 43 (green) days of culture. Results show the average of biological triplicates and 95% confidence limits.

The chemical composition of the wheat–straw treated with *P. eryngii* was analyzed, and compared with control straw. Changes over time in the relative abundance of its main constituents, determined as acetone extractives, water-soluble material, Klason lignin, acid-soluble lignin, and monosaccharides in cellulose, hemicellulose and pectin polymers, are shown in Figure 13A. The fungal growth produced 15.7% weight loss after 43 days as a consequence of a decrease in the content of all these components, each of them to a different extent. Thus, the acetone extracts—including free fatty acids, fatty alcohols, free sterols, steroid ketones, steroid glycosides and waxes among other lipophilic compounds (Figure S5)—experienced a 49% decrease, whereas the water-soluble material suffered a 43% reduction. The decrease of the different lipophilic compounds is most probably due to the activity of some of the over 300 proteins identified in the transcriptome as involved in lipid metabolism and transport, such as up-regulated lipases and P450s (Table S7). Lipases and β-glycosidases would be involved in the decrease of fatty-acid glycerides and glycosides, respectively, while P450s could participate in the catabolism of steroids and fatty acids. Interestingly, an increase of some of the above lipophilic compounds (including W42 and W44 waxes and oleic and linoleic unsaturated fatty acids) was also observed at different stages during the treatment, due to fungal biosynthesis. Different putative fatty-acid desaturases identified in the transcriptome, including two delta-9 acyl-CoA desaturases (one of them, JGI-ID 1394454, highly transcribed) (Table S8), could be responsible for the synthesis of unsaturated fatty acids.

Polysaccharides started to be modified at the initial stages of fungal growth, as demonstrated by the decrease in glucose, xylose, arabinose, mannose, and galactose on day 6. Changes in the acid-soluble lignin, which would include low molecular-weight degradation products [61] were also observed since day 6. By contrast the polymeric lignin content (Klason lignin) changed minimally during the first 6 days of culture, although 4.9% degradation was produced on day 14 and 5.6% on day 43.

Changes in the concentration of some of the most abundant polar aromatics present in the water phase of wheat–straw were analyzed by GC/MS (Figure 13B). These included different free phenolics such as lignin precursors and related aromatic aldehydes and ketones. All these monomeric aromatic compounds were depleted at day 6—which suggests extracellular degradation or modification or intracellular catabolism, as suggested for pHBA in the previous section—except pHBA which was depleted on day 14, and benzoic acid that remained during the whole incubation process.

We also analyzed the presence of different carboxylic acids and their evolution over time in the same water phase of the wheat–straw cultures (Figure S6) due to their capacity to act as Mn^3+^ chelators. The Mn^3+^ chelates would act as diffusible oxidizers of phenolic lignin [62], in addition to initiate lipid peroxidation leading to reactive radicals [63] breaking down non-phenolic lignin model dimers [64]. The levels of malonic, succinic, and fumaric acids in wheat–straw remained relatively stable during the first 6 days of culture, whereas those of malic acid were reduced by half. Then, fumaric and malic acids disappeared, but succinic and malonic acids were still detected on day 14. Although succinic has been shown not to form a complex with Mn^3+^ [65], the presence of malonic acid, an optimal Mn^3+^ chelator [66], would guarantee the activity of those Mn^2+^-oxidizing peroxidases (MnPs and DyP) identified in the extracellular medium.

**Figure 14:**
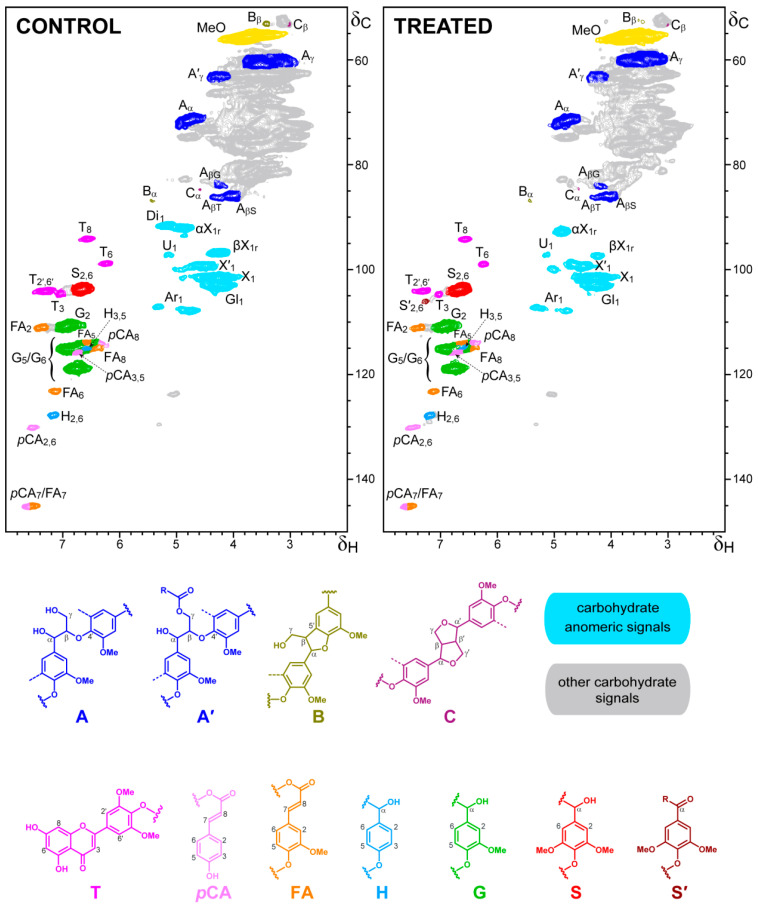
NMR analysis of control (uninoculated) wheat–straw and treated with *P. eryngii* for 43 days, acquired at the gel stage in DMSO-*d*_6_. Top. HSQC spectra displaying ^13^C-^1^H cross-correlation signals of lignin, cinnamic acid, and carbohydrate moieties. Bottom. Lignin and other aromatic structures identified including: **A**, β–*O*–4′ ether substructure (including a second S, G or T unit); **A′**, γ-acylated β-*O*-4′ ether substructure; **B**, phenylcoumaran substructure; **C**, resinol substructure; **T**, tricin substructure; ***p*CA**, *p*-coumaric acid; **FA**, ferulic acid; **H**, *p*-hydroxyphenyl unit, **G**, guaiacyl unit; **S**, syringyl unit; and **S’**, Cα-oxidized S unit (R in S’ can be a hydroxyl in carboxylic acids, a hydrogen in aldehydes, or a lignin side chain in ketones). For carbohydrates, only the anomeric signals were assigned corresponding to: **Ar**, arabinose units; **Di**, glucose moiety in disaccharide; **Gl**, glucose units; **U**, uronic acid units; **X** and **X’**, normal and acetylated xylose units; and **αXr** and **βXr**, α and β xylose reducing units.

A more detailed analysis of changes in lignin, carbohydrates and cinnamic acids during the fungal growth on wheat–straw was performed by 2D-NMR of the whole sample (at the gel state) (Figure 14). Under these conditions, the signals of the lignin *p*-hydroxyphenyl (**H**), guaiacyl (**G**) and syringyl (**S**) units, *p*-coumaric acid (***p*CA**), ferulic acid (**FA**) and tricin (**T**) aromatic structures (in the δ_C_/δ_H_ 90–150/6–8 region of the HSQC spectra) were well resolved without overlapping with carbohydrate signals. In contrast, the signals of lignin side chains characterizing the β–*O*–4′ (**A**), phenylcoumaran (**B**) and resinol (**C**) substructures, identified in the oxygenated aliphatic region of the spectra (δ_C_/δ_H_ 50–90/3–5.5), partially overlapped with different carbohydrate signals concentrated in this region. Fortunately, this is not the case of signals of the carbohydrate anomeric carbons, which occupy an intermediate position (δ_C_/δ_H_ 90–110/3.5–5.5 region) without significant overlapping with other structures. Since the major cellulose fraction is crystalline, and therefore “silent” under the gel-state NMR conditions, the identified anomeric signals mainly correspond to hemicellulosic xylan, arabinan, glucan, and uronic acids.

In general terms, the 2D-NMR analyses did not reveal strong modification of the wheat–straw lignin and cinnamic acids after fungal growth, and the main differences were observed in the carbohydrate anomeric region. These include the decrease of signals of xylose reducing ends (in α and β configurations), suggesting a preferential degradation of oligoxylosaccharides, together with disappearance of the signal at δ_C_/δ_H_ 92/5.2 (assigned to glucose C_1_-H_1_ correlation in wheat–straw disaccharide), and decreases in arabinose signals, among others. In the case of lignin, the oxidative nature of its fungal attack agrees with the appearance of a new signal assigned to the equivalent C_2_-H_2_ and C_6_-H_6_ correlations in Cα-oxidized S units (S’_2,6_ signal, corresponding to terminal aldehydes/carboxylic acids or intercalar ketone units). Moreover, there was a preferential degradation of the G lignin units over the S units during the fungal treatment of wheat–straw, resulting in higher lignin S/G ratio in the treated sample (0.6) than in the control (0.4). Globally, the total amount of lignin units (H, G and S) plus other aromatic structures (such as *p*CA, FA and tricin) in the treated samples was only slightly lower than in the control wheat–straw.

## 4. Discussion

The genome of the monokaryotic *P. eryngii* ATCC 90797 was recently sequenced to obtain insights into its lignocellulolytic capabilities [17]. This study gives a step forward and shows the temporal dynamics of its enzymatic machinery involved in the degradation of non-woody lignocellulose via a multiomic study which includes transcriptome, exoproteome, and metabolome analyses in wheat–straw and glucose–ammonium cultures. The significant differences in the transcriptome patterns at short-time growth (6-d culture) (Figure 2) would be the result of the early adaptation of *P. eryngii* to the lignocellulosic substrate, while the small changes that occurred in long-term wheat–straw culture were interpreted as a nearly complete metabolic adaptation to grow on this substrate.

According to the GO enrichment analysis, the adaptive response to the wheat–straw substrate mainly consisted of up-regulation of genes responsible for: (i) triggering extracellular biological processes involved in the metabolism of carbohydrates, including binding to and hydrolysis of plant cell-wall polysaccharides; (ii) carbohydrate and amino-acid transport; and (iii) ligninolytic peroxidases and P450s involved in lignin degradation, lipid metabolism, and detoxification processes, among others [7,67]. In contrast, no significant improvements were observed in wheat–straw (compared to glucose–ammonium medium) with respect to the expression of hydrophobins (Table S9), which have been proposed to mediate hyphal degradation of hydrophobic substrates such as lignin [54].

The transcriptional adaptation to the lignocellulosic substrate, with up to 545 up-regulated genes (log2-fold change ≥ 2; FDR < 0.05), was also reflected in the exoproteome composition with 897 identified proteins. This number is higher than reported for exoproteomes of other fungi growing on lignocellulosic substrates, which included 118 extracellular proteins for *Phanerochaete chrysosporium*, 72 proteins for *Postia placenta* and 135 proteins for *Pycnoporus coccineus* colonizing aspen and pine wood [19,68]; 121 proteins in filtrates from aspen wood cultures of *C. subvermispora* [11]; 793 proteins for *P. chrysosporium* growing on different poplar genotypes [69]; and 434 proteins for *P. ostreatus* growing on poplar wood and wheat–straw [70]. A higher number, in this case of total proteins (1356 proteins), was only reported for *Phlebia radiata* growing on spruce wood [71]. Elucidating the precise function of many of these proteins in lignocellulose degradation remains a task for future research, and this is of particular interest in the case of *P. eryngii* considering that 160 of the 545 up-regulated genes encode proteins whose functions are completely unknown.

As expected, wheat–straw colonization by *P. eryngii* required a high number and diversity of extracellular proteins compared to those needed to grow using glucose and ammonium as easily assimilated carbon and nitrogen sources. Overall, the composition of *P. eryngii* exoproteome was relatively constant during wheat–straw cultivation, with 41% of the proteins shared at each time point from day 6 to 43. A similar stabilization over time in the set of proteins released to the extracellular medium was previously reported for the ligninolytic fungus *P. radiata* growing on spruce wood for 42 days [71]. A relevant percentage of the extracellular proteins released on this lignocellulosic material were CAZymes and oxidoreductases directly or indirectly involved in plant-cell-wall degradation, peptidases contributing to recycle extracellular organic nitrogen, and proteins with unknown function. Most of them were predicted to be secreted by conventional and unconventional pathways, although release by hyphal lysis was assumed for those enzymes whose presence in the extracellular medium could not be explained in any other way.

Our results show that *P. eryngii* releases an “almost complete” repertoire of enzymes for cellulose, hemicellulose, pectin and lignin degradation at the initial stages of growth on wheat–straw (here day 6). Although GHs, CEs and PLs active on cell-wall polysaccharides are well represented in the exoproteome by both up-regulated and constitutive genes on day 6 (Figure 6 and Figure 9), the repertoire of PCWDEs is not complete since different oxidoreductases with a key role in lignocellulose degradation (PODs, AA9 and AA16 LPMOs, CDHs, and MOXs) are absent from the exoproteome (even if most of them appeared up-regulated in the transcriptome from that day on). This is not common in white-rot fungi, although the absence of PODs in *P. chrysosporium* [69,72] and of different oxidative enzymes for lignin degradation and partner enzymes for H_2_O_2_ production in *P. coccineus* [73] have also been reported at short-term growth on lignocellulosic substrates. The fact that the products of these up-regulated genes are not present in the exoproteome at the early stage of substrate colonization can be justified by the existence of post-transcriptional regulations.

Interestingly, while PODs were not detected at the early stages of wheat–straw degradation, laccases appeared well represented. LAC JGI-ID 1449727 was constitutively expressed and abundantly identified on wheat–straw until day 43. The most abundant laccase in the exoproteome, however, was NLAC (JGI-ID 1521536). This enzyme corresponds to LacII characterized by Muñoz et al. [32] (as revealed by the Nt sequence: ATKKLDFHIRN), which is induced by lignin and vanillic acid [74] present in wheat–straw (Figure 13B). Laccases are able to oxidize phenolic lignin units and *p*-hydroxycinnamic acids (also present in wheat–straw, Figure 13), forming phenoxyl radicals that have been suggested to act as mediators in lignin degradation [75]. These enzymes could also initiate lipid peroxidation reactions (we identified oleic and linoleic acid in the wheat–straw samples, Figure S5) in the presence of *p*-hydroxycinnamic acids [76]. In consequence, the laccases identified in the secretome could be responsible for the small changes observed in acid-soluble and polymeric lignin (Klason lignin) on day 6 (Figure 13A).

Surprisingly, high amounts of constitutively expressed AAOs, and to a lesser extent up-regulated and constitutive CROs, were observed in the extracellular medium from day 6. In fact, AAOs were by far the most abundant extracellular proteins identified in the *P. eryngii* exoproteome. This has not been described before in any other ligninolytic fungus, including related *P. ostreatus* growing on woody and non-woody lignocellulosic substrates [70]. AAOs play an important role in extracellular H_2_O_2_ production by *P. eryngii* from aromatic alcohols, aldehydes and acids synthesized by the fungus, and also identified here in wheat–straw (Figure 13) [60]. The participation of mycelium-associated dehydrogenases in this redox-cycling has been demonstrated [77], although the corresponding genes were unknown. Dehydrogenases with this putative role were identified as highly transcribed and up-regulated enzymes on days 6 and 14 (Figure 12, enzymatic steps 12 and 14) indicating that they could contribute to guarantee a continuous supply of H_2_O_2_ by AAOs throughout fungal growth. In turn, H_2_O_2_ is a strong inducer of ligninolytic peroxidase expression in *P. eryngii* [78]. Therefore, peroxide generated by AAOs (and CROs) could be one of the main factors responsible for the up-regulation of ligninolytic peroxidases observed at the transcriptome level on days 6 and 14, which would translate into the MnPs and VPs identified in the exoproteome on days 14 and 43. This analysis suggests a relevant role for AAOs providing H_2_O_2_ both for inducing the expression of ligninolytic peroxidases and as a co-substrate of these enzymes, among other processes.

After the early adaptation period of *P. eryngii* to wheat–straw, the enzymatic set released to the extracellular medium for cellulose, hemicellulose, and pectin depolymerization increased in the number and abundance of GHs, CEs, and PLs in long-term cultivation. These included a high number of CBM1-containing PCWDEs from key-families such as GH6 and GH7 active on cellulose, GH10 and GH11 xylanases, and PL3 active on pectin (Figure 10). The appearance of oxidoreductases active on cellulose in the exoproteome on days 14 and 43 contributed to increase the cellulolytic potential of the fungus. Among others, *P. eryngii* released nine AA9 LPMOs and one AA16 LPMO (as up-regulated enzymes, most of them also highly transcribed) active on crystalline cellulose; and one up-regulated CDH (Figure 7) being able to activate the LPMOs [79], among other possible roles [80]. In a recent genomic study, the expansion of genes encoding AA9 LPMOs together with the increase in CBM1-appended PCWDEs were presented as outstanding evolutionary events in grass-litter decomposers [17]. Our results confirm this also at secretomic level in *P. eryngii*, taking into account the high content of AA9 LPMOs and CBM1-appended PCWDEs produced by this fungus on wheat–straw, in comparison with the wood decomposer *P. ostreatus* when grown on both poplar wood and wheat–straw [70].

The first ligninolytic peroxidases were also identified in the wheat–straw exoproteome of *P. eryngii* on day 14. The *P. eryngii* genome includes 15 peroxidase genes corresponding to eight PODs classified as AAs, four DyPs, and three UPOs [17]. The eight PODs have been identified as: (i) three VPs, including the first enzyme ever characterized from this family (JGI-ID 1429886) [81], which is able to oxidize phenolic and non-phenolic lignin [82]; and (ii) five manganese peroxidases of the short-MnP subfamily (including the only enzyme of this subfamily characterized to date in this species [83]), its members eventually showing Mn-independent activities [20]. This set of PODs is practically identical to that characterized from *P. ostreatus* [20] (*P. eryngii* has one less MnP). The five MnPs and one VP were identified in the exoproteome of *P. eryngii* as minor proteins (VP only at day 43) together with a DyP homologous to *P. ostreatus* DyP4, which is the only fungal DyP described to date able to oxidize Mn^2+^ to Mn^3+^, as MnPs and VPs do [84]. By contrast, we did not identified any UPO that could support the putative contribution of these enzymes to lignin degradation, as previously suggested for the *Agrocybe aegerita* enzyme [85]. This enzymatic configuration and the presence of malonic acid in the extracellular medium (Figure S6), which is considered to be an optimal Mn^3+^ chelator [66], are compatible with a ligninolytic activity mediated by peroxidases oxidizing Mn^2+^ (MnPs and DyP), together with the putative contribution of laccases previously described. The H_2_O_2_ formation necessary for peroxidase activity should be guaranteed by the peroxide-producing enzymes identified. The presence of MOX in the extracellular medium suggests H_2_O_2_ production associated with lignin demethylation [86], together with H_2_O_2_ generated by the highly secreted AAOs (and CROs) from day 6.

Different studies pinpointed *P. eryngii* as a species of biotechnological interest [87] due to its ability to grow and transform non-woody lignocellulosic substrates in applications such as biological pulping for paper manufacturing [28] or biofuel production [29]. Wheat–straw has been the most investigated lignocellulosic substrate for growing *P. eryngii* under solid-state fermentation conditions, and its chemical modification has been analyzed [88,89] but a comprehensive analysis of the enzymatic machineries involved, using modern multiomic approaches, was lacking. The small differences between the ligninolytic capabilities observed in our study and those previously reported for *P. eryngii* [88] could be due to differences in the physiological development of monokaryotic and dikaryotic strains as previously suggested by Echelerová et al. [90] for *P. ostreatus*. The results presented here confirm previous studies reporting that wheat–straw transformation by *P. eryngii* proceeds with a very moderate loss of substrate weight, compared to other white-rot fungi. In this way, Valmaseda et al. [88] reported only 7% loss of weight in solid-state fermentation of wheat–straw with this fungus (although with a greater degradation of lignin of that observed in our study), compared with 46% loss with *P. chrysosporium* and 56% loss with *T. versicolor*. Moreover, related studies have shown the existence of substrate-dependent degradation patterns in the decay of straw and wood by basidiomycetes [91,92] with *P. eryngii* appearing as one of the most promising species for transformation of non-woody lignocellulosic substrates.

Finally, not only plant cell-wall polysaccharides are used as carbon and energy sources by white-rot basidiomycetes. Recently, del Cerro et al. [49] described intracellular pathways for the catabolism of lignin-derived aromatic compounds in two of these fungi and determined the main enzymes involved in those pathways. Based on that information, we searched for homologous enzymes in *P. eryngii* and found numerous matches. In view of these results, we hypothesize that *P. eryngii*, similarly to *T. versicolor* and *C. subvermispora* [49], may be able to use monomeric aromatic compounds as carbon sources, which enter central carbon metabolism via acetyl-CoA and succinyl-CoA, although obviously this hypothesis will require further experimental confirmation, for instance, via targeted metabolomics or isotopic-labeling studies.

Overall, this study combines chemical analyses of wheat–straw—which include state-of-the-art 2D-NMR spectroscopy to study lignocellulose modification—with genomic, transcriptomic, and proteomic analyses to unveil the complex enzymatic machinery that *P. eryngii* activates to decompose the different plant cell-wall polymers constituting its carbon and energy source. This comprehensive dataset is not only key towards better understanding the metabolism of this lignin-degrading organism but also to identify enzymes and enzyme cocktails that could be deployed in processes for the effective conversion of lignocellulose to valuable products.

## Supplementary Materials

The following are available online at https://www.mdpi.com/article/10.3390/jof7060426/s1. Figure S1: Log2 normalized RNA-seq read counts of 18 housekeeping genes. Genes for chitin synthases, NADH-ubiquinone oxidoreductases and tubulin are shown at 6, 14, and 43 days of culture on two substrates. WS/G-A: Wheat–straw/Glucose–ammonium medium. d6/d14: 6/14 days of culture. Figure S2: Pearson’s Correlation (PC) Heatmap of the RNA-seq counts for each pair of libraries, grouped by growth conditions. WS/G-A: Wheat–straw, Glucose–ammonium medium. 6/14: 6/14 days of culture. Figure S3: Functional distribution of proteins identified in the exoproteome of *P. eryngii* grown on glucose–ammonium medium. (A) Venn diagram of total protein numbers (189) after 6 (blue), 14 (red) and 43 (green) days of culture. (B) Distribution into functional groups. (C) Distribution of CAZymes and oxidoreductases on 6, 14, and 43 days of culture. The number of proteins is followed by their percentage in B and C, and their predicted functions are indicated by color codes. Figure S4: Comparison of the abundance in PSM values of the main protein types, indicated by color codes, in the exoproteome on glucose–ammonium medium after 6, 14, and 43 days of incubation. The central numbers correspond to the secreted proteins (left) and their abundance in PSM values (right). Black and yellow arcs indicate oxidoreductases and CAZymes, respectively. Figure S5: Lipid composition (mg/g) from wheat–straw before inoculation (control) with *P. eryngii* and after 6, 14, and 43 days of culture. Figure S6: (A) Concentration of carboxylic compounds in the wheat–straw soluble fraction before inoculation (black), and after 6 (blue) and 14 (red) days of culture with *P. eryngii*. Results show the average of biological triplicates and 95% confidence limits. Table S1: Proteins (JGI-IDs) encoded in the genome of *P. eryngii*, identified in the transcriptome and exoproteome of this fungus grown on wheat–straw and in glucose–ammonium medium (control) after 6, 14, and 43 days of culture. The table includes log2 normalized RNA-seq read counts (averaged from the replicates at all growth points) and shows fold-change differences in gene expression between substrates (wheat–straw vs. glucose–ammonium) (statistical significance, Wald test FDR < 0.05). Up-regulated genes are represented with a green arrow, and highly transcribed genes with a green circle. Up-regulated genes statistically validated are shown over gray–blue background whereas non-validated genes are shown over red background in columns “FDR day 6” and “FDR day 14”. Exoproteome information is represented as PSM values. The theoretical secretome consists of proteins secreted by the conventional (SECRETOOL) and unconventional (ApoplastP) pathways. If conventional or unconventional secretion are not indicated for a protein, its presence in the theoretical secretome is represented as “NO”. Expert functional annotations show CAZymes and oxidoreductases (AAs) with their respective links to www.cazy.org, as well as whether they are PCWDE (plant cell-wall degrading enzymes) or FCWDE (fungal cell-wall degrading enzymes), and on which polymer they act. Gene functional annotations according to Gene Ontology (GO), InterPro (IPR), Kyoto Encyclopedia of Genes and Genomes (KEGG), EuKaryotic Orthologous Groups (KOG) and Enzyme Commission numbers (EC number) are indicated. Table S2: List of reference proteins for aromatic catabolism in *T. versicolor* and *C. subvermispora*, and corresponding hits in *P. eryngii*. The activity of some of the selected enzymes in *T. versicolor* and *C. subvermispora* was tested and validated in the presence of different substrates [49]. pHBA = 4-hydroxybenzoic acid, HQ = hydroquinone. Table S3: Repertoire of glycoside hydrolase (GH), polysaccharide lyase (PL), carbohydrate esterase (CE) and glycosyl transferase (GT) CAZymes; oxidoreductases classified as Auxiliary Activities (AA) in CAZy database (www.cazy.org); unspecific peroxygenases (UPO); dye-decolorizing peroxidases (DyP); carbohydrate-binding modules (CBM); and hydrophobins in the *P. eryngii* genome. Protein reference numbers, JGI-IDs, are available at the JGI fungal genome portal MycoCosm through https://mycocosm.jgi.doe.gov/Pleery1. Table S4: Gene Ontology enrichment analysis for up-regulated genes on wheat–straw vs. glucose–ammonium medium. Identification, transcriptomic regulation, and functions of proteins that are part of the enriched GO terms classified according to the Biological Process in which they are involved (BP), Cellular Component where they are localized (CC) and Molecular Function (MF). The table includes: JGI protein IDs; fold change of gene expression between substrates (wheat–straw and glucose–ammonium) (statistical significance, Wald test FDR < 0.05); functional annotation according to CAZy database; PCWDE, plant cell-wall degrading enzyme, FCWDE, fungal cell-wall degrading enzyme; substrate on which the enzymes act; and automatic annotations of EuKaryotic Orthologous Groups (KOG). Table S5: Exclusively highly transcribed CAZymes, oxidoreductases (AA) and CBMs identified in the wheat–straw exoproteome. The enzymes are sorted from highest to lowest abundance based on total PSM values. The protein reference numbers (JG-ID#) correspond to those in the catalog of predicted proteins from the *P. eryngii* ATCC 90797 genome available through JGI MycoCosm Genome Portal. Protein references over yellow background correspond to enzymes exclusively secreted on wheat–straw. The presence (+) or absence (-) of theoretical secretion (T.S) is indicated. Symbol + in blue or orange color represents proteins predicted to be secreted by the classical secretory pathway or by the unconventional pathway, respectively. Colors in the (sub)family column correspond to: GH, green; oxidoreductases, yellow; CE, purple; GT, blue; and CBM, gray. Table S6: Proteins predicted in *P. eryngii* for aromatics catabolism (see Table S2), including information about: (i) the proposed enzymatic steps for 4-hydroxybenzoic acid (pHBA) conversion in which they would be involved; (ii) their presence in the theoretical secretome (obtained using SECRETOOL and ApoplastP as described in the main manuscript); (iii) their expression at transcriptome and exoproteome level (up-regulated genes are represented with a green arrow, and highly transcribed genes with a green circle); and (iv) functional annotation. Table S7: Proteins putatively involved in lipid transport and metabolism identified in the transcriptome and exoproteome of *P. eryngii* grown on wheat–straw and in glucose–ammonium medium. The table includes log2 normalized RNA-seq read counts (averaged from the replicates at all growth points) and shows fold-change differences in gene expression between substrates (wheat–straw vs. glucose–ammonium) (statistical significance, Wald test FDR < 0.05). Up-regulated genes are represented with a green arrow and highly transcribed genes with a green circle. Up-regulated genes statistically validated are shown over gray–blue background while non-validated genes are shown over red background in columns “FDR day 6” and “FDR day 14”. Exoproteome information is represented as PSM values. The theoretical secretome consists of proteins secreted by the conventional (SECRETOOL) and unconventional (ApoplastP) pathways. If conventional or unconventional secretion are not indicated for a protein, its presence in the theoretical secretome is represented as “NO”. Gene functional annotations according to Gene Ontology (GO), InterPro (IPR), Kyoto Encyclopedia of Genes and Genomes (KEGG), EuKaryotic Orthologous Groups (KOG) and Enzyme Commission numbers (EC number) are indicated. Table S8: Enzymes with putative fatty-acid desaturase activity identified in the transcriptome of *P. eryngii* grown on wheat–straw and in glucose–ammonium medium. The table includes log2 normalized RNA-seq read counts (averaged from the replicates at all growth points). Fold-change differences in gene expression between substrates (wheat–straw vs. glucose–ammonium) (statistical significance, Wald test FDR < 0.05) are not observed. Genes with log2(counts) ≥ 12 were considered highly transcribed, and they appear represented with a green circle. The enzymes are not present in the theoretical secretome nor in the exoproteome (PSM values = 0). Gene functional annotations according to Gene Ontology (GO), InterPro (IPR), Kyoto Encyclopedia of Genes and Genomes (KEGG), EuKaryotic Orthologous Groups (KOG) and Enzyme Commission numbers (EC number) are indicated. Table S9: Hydrophobins (JGI identification numbers, JGI-IDs) encoded in the genome of *P. eryngii*. They were identified in the transcriptome of *P. eryngii* grown on wheat–straw and in glucose–ammonium medium (control) after 6 and 14 days of culture. The table includes log2 normalized RNA-seq read counts (averaged from the replicates at all growth points) and shows fold-change differences (FC) in gene expression between substrates (wheat–straw vs. glucose–ammonium) (statistical significance, Wald test FDR < 0.05). Hydrophobins did not appeared in the exoproteome (PSM values = 0).
