# Supplementary material for: A Multiomic Approach to Understand How Pleurotus eryngii Transforms Non-Woody Lignocellulosic Material
Source: J Fungi (Basel). 2021 May 28;7(6):426. doi: 10.3390/jof7060426 (PMC8227661; doi:10.3390/jof7060426)
Supplement: Supplementary file 1 [file jof-07-00426-s001.zip › Supplementary_Material/Suppl_Figs_S1-S6.pdf]

Figure S1

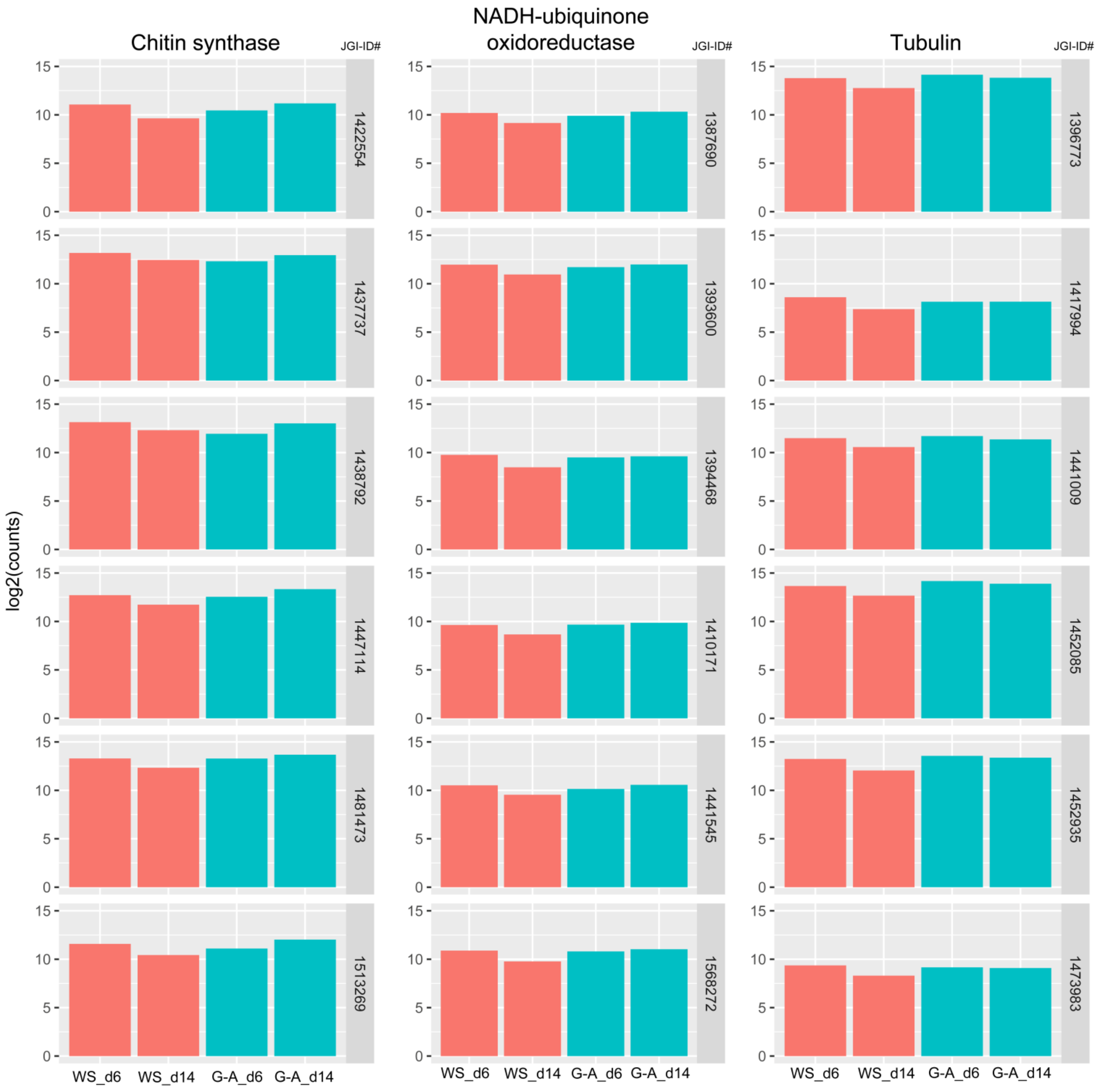

Figure S1: Log2 normalized RNA-seq read counts of 18 housekeeping genes. Genes for chitin synthases, NADH-ubiquinone oxidoreductases and tubulin are shown at 6, 14 and 43 days of culture on two substrates. WS/G-A: Wheat straw/Glucose-ammonium medium. d6/d14: six/fourteen days of culture.

Figure S2

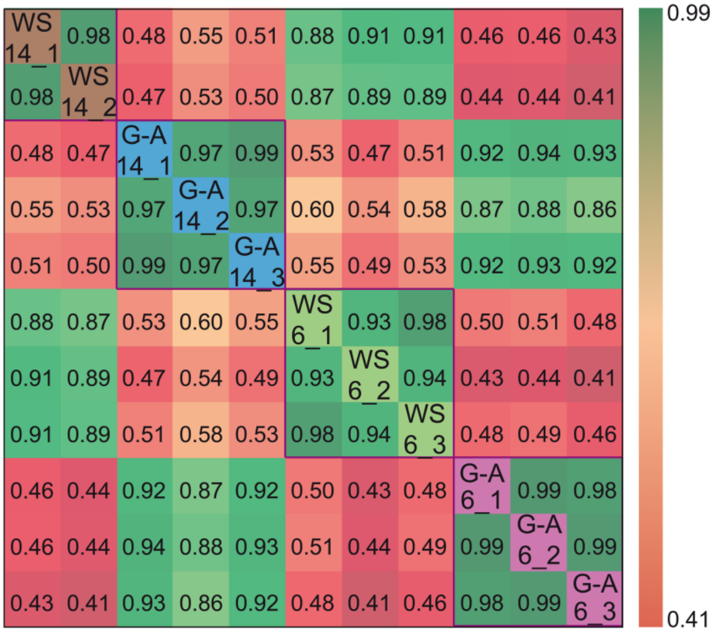

Figure S2: Pearson's Correlation (PC) Heatmap of the RNA-seq counts for each pair of libraries, grouped by growth conditions. WS/G-A: Wheat straw/Glucose-ammonium medium. 6/14: six/fourteen days of culture.

Figure S3

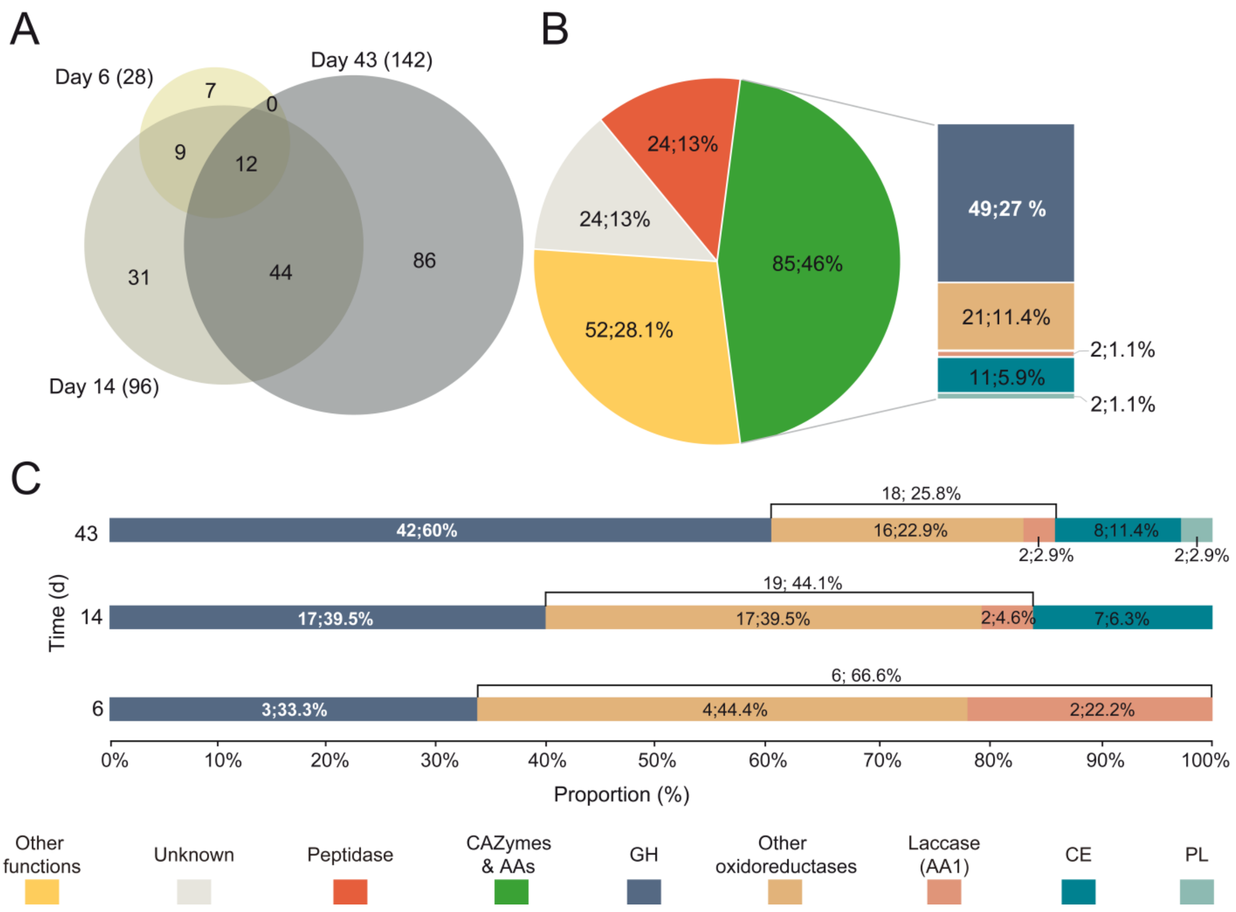

Figure S3. Functional distribution of proteins identified in the exoproteome of *P. eryngii* grown on glucose-ammonium medium. (A) Venn diagram of total protein numbers (189) after 6 (blue), 14 (red) and 43 (green) days of culture. (B) Distribution into functional groups. (C) Distribution of CAZymes and oxidoreductases on 6, 14 and 43 days of culture. The number of proteins is followed by their percentage in B and C, and their predicted functions are indicated by color codes.

Figure S4

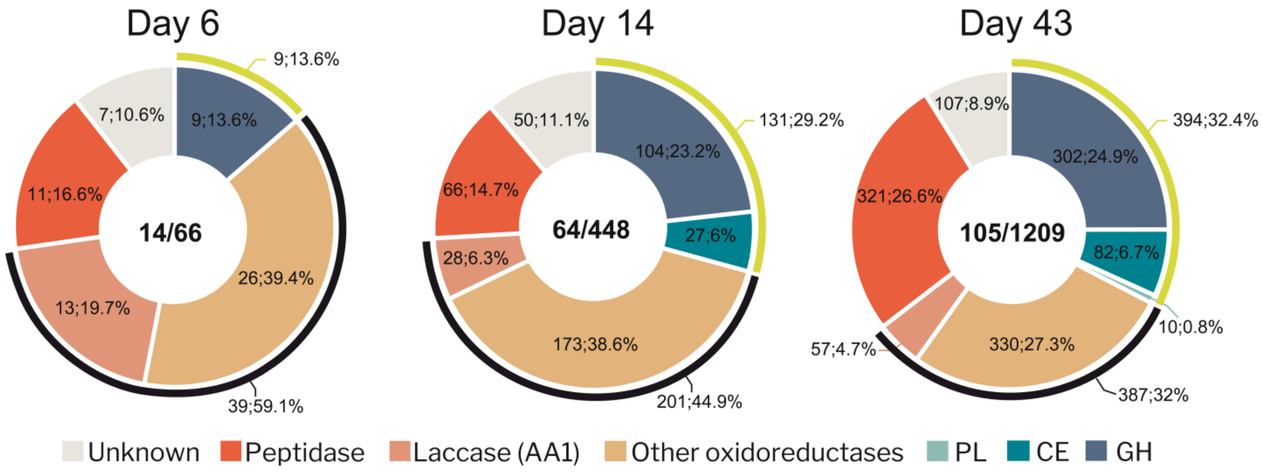

Figure S4. Comparison of the abundance in PSM values of the main protein types, indicated by color codes, in the exoproteome on glucose-ammonium medium after 6, 14 and 43 days of incubation. The central numbers correspond to the secreted proteins (left) and their abundance in PSM values (right). Black and yellow arcs indicate oxidoreductases and CAZymes, respectively.

Figure S5

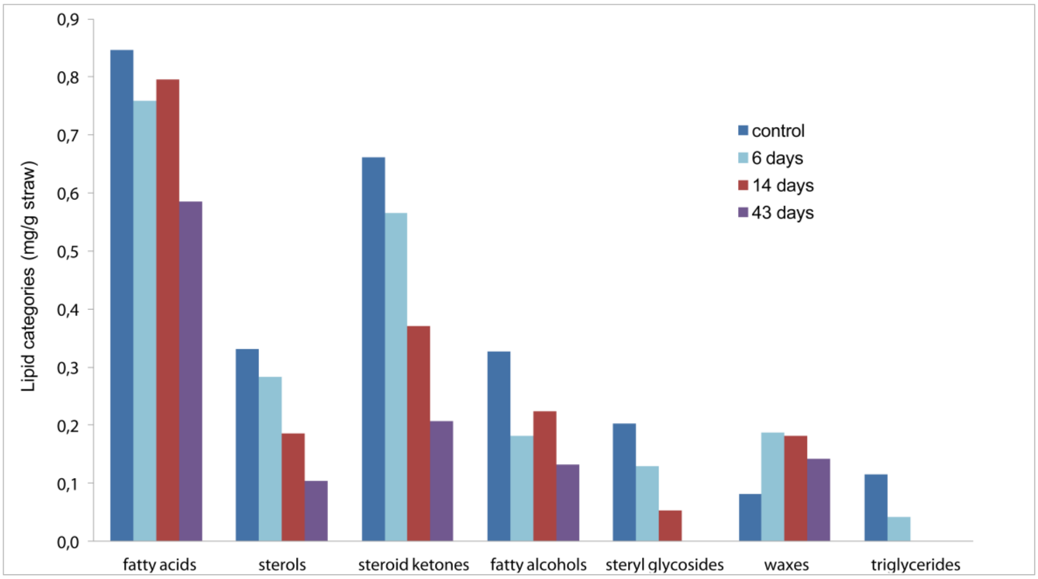

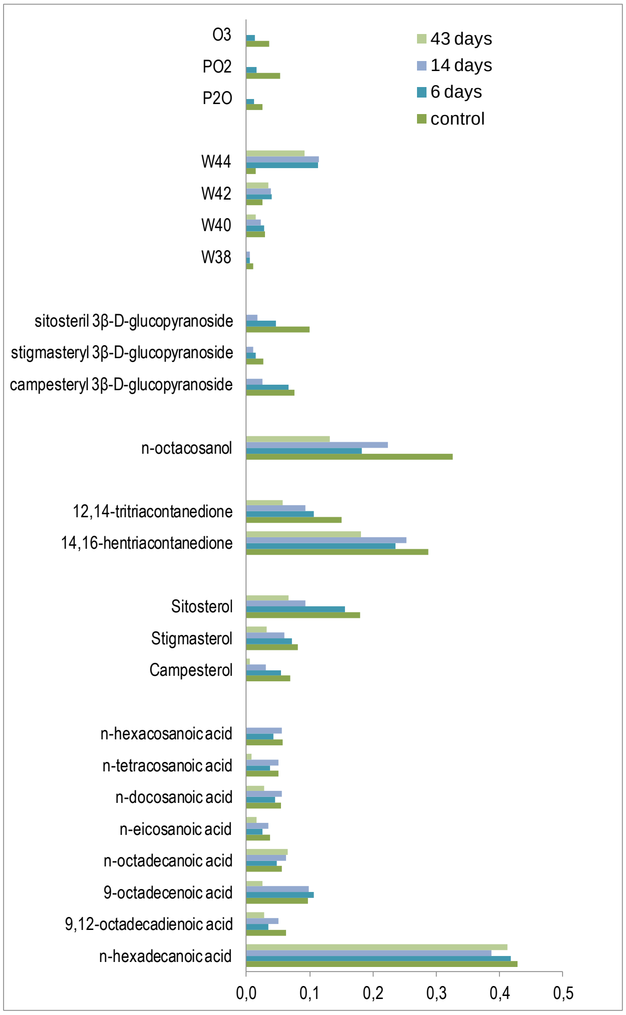

Figure S5. Lipid composition (mg/g) from wheat straw before inoculation (control) with *P. eryngii* and after 6, 14 and 43 days of culture.

Figure S6

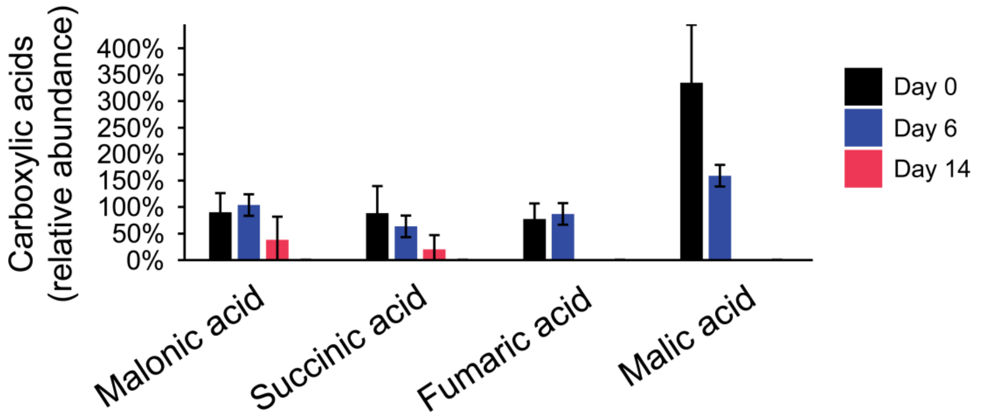

Figure S6. (A) Concentration of carboxylic compounds in the wheat straw soluble fraction before inoculation (black), and after 6 (blue) and 14 (red) days of culture with *P. eryngii*. Results show the average of biological triplicates and 95% confidence limits.
