# Supplementary material for: A Multiomic Approach to Understand How Pleurotus eryngii Transforms Non-Woody Lignocellulosic Material
Source: J Fungi (Basel). 2021 May 28;7(6):426. doi: 10.3390/jof7060426 (PMC8227661; doi:10.3390/jof7060426)
Supplement: Supplementary file 1 [file jof-07-00426-s001.zip › Supplementary_Material/Table S2.pdf]

Table S2. List of reference proteins for aromatic catabolism in *T. versicolor* and *C. subvermispora*, and corresponding hits in *P. eryngii*. The activity of some of the selected enzymes in *T. versicolor* and *C. subvermispora* was tested and validated in the presence of different substrates [1]. pHBA= 4-hydroxybenzoic acid, HQ= hydroquinone.

Reference protein IDs	Enzyme activity, substrate utilized for validation	Hits in *P. eryngii* (protein ID)	% Identity and e-values of the hits
CERSUDRAFT_120062	Oxidative decarboxylase, validated on pHBA and protocatechuate	Pleery1_1521705<br>Pleery1_1510672	57.3%, 3.5e-178<br>52.7%, 2.2e-163
CERSUDRAFT_90429	Oxidative decarboxylase, validated on pHBA	Pleery1_1437817	46.6%, 9.4e-134
TRAVEDRAFT_32834	Oxidative decarboxylase, validated on protocatechuate	Pleery1_1437817	44.4%, 8.0e-121
TRAVEDRAFT_58730	Hydroxylase, validated on pHBA and HQ	Pleery1_1471020	60.9%, 0.0
CERSUDRAFT_82057	Hydroxylase, validated on HQ	Pleery1_1471020	64.5%, 0.0
TRAVEDRAFT_28066	Dioxygenase	Pleery1_1440135	64.6%, 5.5e-131
CERSUDRAFT_116134	Dioxygenase	Pleery1_1440135	66.7%, 1.1e-151
TRAVEDRAFT_129211	Cytochrome P450, not validated (putative conversion of pHBA to protocatechuate)	Pleery1_1484875<br>Pleery1_1392807	65.8%, 0.0<br>40.3%, 2.1e-107
CERSUDRAFT_116910	Cytochrome P450, not validated (putative conversion of pHBA to protocatechuate)	Pleery1_1484875<br>Pleery1_1392807	68.3%, 0.0<br>40.4%, 1.2e-112
TRAVEDRAFT_26150	Oxidoreductase, not validated (putative conversion of 4-hydroxymuconate semialdehyde to maleylacetate)	Pleery1_1456897<br>Pleery1_1436180	65.9%, 0.0<br>55.8%, 0.0
CERSUDRAFT_139148	Oxidoreductase, not validated (putative conversion of 4-hydroxymuconate semialdehyde to maleylacetate)	Pleery1_1456897<br>Pleery1_1436180	64.9%, 0.0<br>59.1%, 0.0
TRAVEDRAFT_113954	Maleylacetate reductase, not validated (putative conversion of maleylacetate to β-ketoadipate)	Pleery1_1550397	68.0%, 0.0
TRAVEDRAFT_145135	Oxoacid CoA transferase, not validated (putative conversion of β-ketoadipate to β-ketoadipyl CoA)	Pleery1_1380612	72.3%, 0.0
CERSUDRAFT_113511	Oxoacid CoA transferase, not validated (putative conversion of β-ketoadipate to β-ketoadipyl CoA)	Pleery1_1380612	65.8%, 0.0
TRAVEDRAFT_116179	CoA thiolase, not validated (putative conversion of β-ketoadipyl CoA to succinate and acetyl-CoA)	Pleery1_1443243	82.1%. 0.0
CERSUDRAFT_81537	CoA thiolase, not validated (putative conversion of β-ketoadipyl CoA to succinate and acetyl-CoA)	Pleery1_1491356<br>Pleery1_1438132	73.4%, 0.0<br>53.0%, 2.6e-140
TRAVEDRAFT_61111	Oxidoreductase (carboxylic acid reductase), not validated (putative conversion of pHBA to 4-hydroxybenzaldehyde)	Pleery1_1387871<br>Pleery1_1431563	37.6%, 0.0<br>37.9%, 0.0
TRAVEDRAFT_155039	Oxidoreductase (carboxylic acid reductase), not validated (putative conversion of pHBA to 4-hydroxybenzaldehyde)	Pleery1_1387871<br>Pleery1_1431563	37.8%. 0.0<br>37.8%, 0.0
TRAVEDRAFT_170215	Oxidoreductase (carboxylic acid reductase), not validated (putative conversion of pHBA to 4-hydroxybenzaldehyde)	Pleery1_1387871<br>Pleery1_1387810	38.2%, 0.0<br>36.4%, 0.0
CERSUDRAFT_120461	Aldehyde dehydrogenase, not validated (putative conversion of 4-hydroxybenzaldehyde to pHBA)	Pleery1_1456897<br>Pleery1_1436180	66.7%, 0.0<br>56.0%, 0.0
CERSUDRAFT_139148	Aldehyde dehydrogenase, not validated (putative conversion of 4-hydroxybenzaldehyde to pHBA)	Pleery1_1456897<br>Pleery1_1436180	64.9%, 0.0<br>59.1%, 0.0
CERSUDRAFT_114803	Alcohol dehydrogenase, not validated (putative conversion of 4-hydroxybenzaldehyde to 4-hydroxybenzyl alcohol)	Pleery1_1446680<br>Pleery1_1409466	63.6%, 0.0<br>64.5%, 0.0

CERSUDRAFT_116891	Alcohol dehydrogenase, not validated (putative conversion of 4-hydroxybenzaldehyde to 4-hydroxybenzyl alcohol)	Pleery1_1446680<br>Pleery1_1512574	63.8%, 1.8e-109<br>65.9%, 1.5e-106
CERSUDRAFT_116883	Alcohol dehydrogenase, not validated (putative conversion of 4-hydroxybenzaldehyde to 4-hydroxybenzyl alcohol)	Pleery1_1446682<br>Pleery1_1409466	66.5%, 9.7e-177<br>61.7%, 1.1e-176
TRAVEDRAFT_47268	Alcohol dehydrogenase, not validated (putative conversion of 4-hydroxybenzaldehyde to 4-hydroxybenzyl alcohol)	Pleery1_1426281<br>Pleery1_1556441	62.2%, 1.3e-164<br>62.5%, 2.2e-148
TRAVEDRAFT_170422	Alcohol dehydrogenase, not validated (putative conversion of 4-hydroxybenzaldehyde to 4-hydroxybenzyl alcohol)	Pleery1_1505848<br>Pleery1_1420315	63.5%, 1.3e-151<br>63.1%, 2.7e-151
TRAVEDRAFT_167157	Alcohol oxidase, not validated (putative conversion of 4-hydroxybenzyl alcohol to 4-hydroxybenzaldehyde)	Pleery1_319386<br>Pleery1_1435398	50.8%, 0.0<br>48.2%, 0.0
TRAVEDRAFT_157221	Aldehyde oxidase, not validated (putative conversion of 4-hydroxybenzaldehyde to pHBA)	Pleery1_1450186	42.2%, 1.0e-132
TRAVEDRAFT_175111	Aldehyde oxidase, not validated (putative conversion of 4-hydroxybenzaldehyde to pHBA)	Pleery1_1429945<br>Pleery1_1405413	42.9%, 5.9e-124<br>39.0%, 5.4e-117
CERSUDRAFT_152302	Methyltransferase, not validated (putative reversible conversion of 4-hydroxybenzoate to 4-methoxybenzoate)	Pleery1_1507445	43.0%, 1.08e-108
CERSUDRAFT_87325	Methyltransferase, not validated (putative reversible conversion of 4-hydroxybenzoate to 4-methoxybenzoate)	Pleery1_1507445<br>Pleery1_1428432	46.4%, 4.3e-120<br>42.2%, 1.9e-118
CERSUDRAFT_112014	Methyltransferase, not validated (putative reversible conversion of 4-hydroxybenzoate to 4-methoxybenzoate)	Pleery1_1507445<br>Pleery1_1428432	42.9%, 1.4e-107<br>40.0%, 2.0e-103
CERSUDRAFT_114284	Methyltransferase, not validated (putative reversible conversion of 4-hydroxybenzoate to 4-methoxybenzoate)	Pleery1_1507445	42.6%, 1.7e-104

1. Del Cerro, C.; Erickson, E.; Dong, T.; Wong, A.R.; Eder, E.K.; Purvine, S.O.; Mitchell, H.D.; Weitz, K.K.; Markillie, L.M.; Burnet, M.C.; et al. Intracellular pathways for lignin catabolism in white-rot fungi. *Proc. Natl. Acad. Sci. U. S A* **2021**, *118*.
