# Supplementary material for: A Multiomic Approach to Understand How Pleurotus eryngii Transforms Non-Woody Lignocellulosic Material
Source: J Fungi (Basel). 2021 May 28;7(6):426. doi: 10.3390/jof7060426 (PMC8227661; doi:10.3390/jof7060426)
Supplement: Supplementary file 1 [file jof-07-00426-s001.zip › Supplementary_Material/Table S5.pdf]

Table S5

No.	JGI-ID#	T.S	Function	PSM (HT) Day 6	Day 14	Day 43
1	1511425	+	CRO2 (AA5_1)	40 (+)	10 (+)	2
2	1526800	+	GH20	0 (+)	20 (-)	12
3	1450142	+	GH43	7 (-)	14 (+)	11
4	1466492	+	GH72-CBM43	9 (+)	9 (+)	12
5	1452463	+	BQR (AA6)	7 (+)	10 (+)	10
6	1445894	+	CBM13	11 (-)	5 (+)	5
7	1386374	+	CE4	6 (-)	6 (+)	7
8	1428034	+	CBM13	0 (+)	3 (+)	11
9	1499596	-	GT35	0 (+)	2 (+)	8
10	1479939	-	GT4	0 (+)	4 (+)	6
11	1445000	-	CBM12	0 (-)	2 (+)	2
12	1479769	+	GH5_9	0 (+)	4 (-)	0
13	1461766	-	GH5_30	0 (+)	4 (-)	0

Table S5. Exclusively highly transcribed CAZymes, oxidoreductases (AA) and CBMs identified in the wheat straw exoproteome. The enzymes are sorted from highest to lowest abundance based on total PSM values. The protein reference numbers (JG-ID#) correspond to those in the catalog of predicted proteins from the *P. eryngii* ATCC 90797 genome available through JGI MycoCosm Genome Portal. Protein references over yellow background correspond to enzymes exclusively secreted on wheat straw. The presence (+) or absence (-) of theoretical secretion (T.S) is indicated. Symbol + in blue or orange color represents proteins predicted to be secreted by the classical secretory pathway or by the unconventional pathway, respectively. Colors in the (sub)family column correspond to: GH, green; oxidoreductases, yellow; CE, purple; GT, blue; and CBM, gray.
