# Supplementary material for: A Multiomic Approach to Understand How Pleurotus eryngii Transforms Non-Woody Lignocellulosic Material
Source: J Fungi (Basel). 2021 May 28;7(6):426. doi: 10.3390/jof7060426 (PMC8227661; doi:10.3390/jof7060426)
Supplement: Supplementary file 1 [file jof-07-00426-s001.zip › Supplementary_Material/Table S9.pdf]

Table S9

JGI-ID		RNA seq (log2)									
		Day 6					Day 14				
		Wheat straw	Control	FC	FDR	PSMs	Wheat straw	Control	FC	FDR	PSMs
1444721		11.75	12.34	-0.59	1	0	11.20	12.66	-1.46	1	0
1473830		10.57	11.85	-1.28	1	0	9.24	11.91	-2.67	1	0
1068526		10.30	12.34	-2.04	1	0	10.09	12.18	-2.08	1	0
1433269		8.94	7.76	1.18	1	0	8.72	8.46	0.27	1	0
416035		8.83	8.27	0.56	1	0	8.73	8.22	0.51	1	0
1447792		7.47	7.37	0.10	1	0	7.98	7.90	0.08	1	0
1508330		7.41	6.85	0.56	1	0	7.68	7.01	0.67	1	0
1399739		7.26	5.98	1.28	1	0	7.14	6.57	0.56	1	0
809199	Fungal hydrophobin	6.94	7.36	-0.41	1	0	7.60	8.22	-0.62	1	0
1384790		6.80	5.48	1.32	1	0	6.91	5.84	1.08	1	0
833829		6.42	4.88	1.54	1	0	6.96	5.28	1.68	1	0
1509832		6.09	6.40	-0.32	1	0	6.36	6.49	-0.13	1	0
1398020		5.83	4.98	0.85	1	0	6.25	4.87	1.38	1	0
1449904		5.75	3.95	1.8	1	0	4.56	4.2	0.36	1	0
1400791		5.6	7.73	-2.13	0.39	0	5.51	8.89	-3.38	1.5E-11	0
1587123		5.33	4.58	0.76	1	0	5.5	4.6	0.89	1	0
1445631		4.35	3.68	0.67	1	0	4.6	3.95	0.66	1	0

Table S9. Hydrophobins (JGI identification numbers, JGI-IDs) encoded in the genome of *P. eryngii*. They were identified in the transcriptome of *P. eryngii* grown on wheat straw and in glucose ammonium medium (control) after 6 and 14 days of culture. The table includes log2 normalized RNA-seq read counts (averaged from the replicates at all growth points) and shows fold-change differences (FC) in gene expression between substrates (wheat straw *vs* glucose-ammonium) (statistical significance, Wald test FDR < 0.05). Hydrophobins did not appeared in the exoproteome (PSM values =0).
